# Analysis of the Key Elements of FFAT-Like Motifs Identifies New Proteins That Potentially Bind VAP on the ER, Including Two AKAPs and FAPP2

**DOI:** 10.1371/journal.pone.0030455

**Published:** 2012-01-19

**Authors:** Veronika Mikitova, Timothy P. Levine

**Affiliations:** Department of Cell Biology, University College London Institute of Ophthalmology, London, United Kingdom; Virginia Commonwealth University, United States of America

## Abstract

**Background:**

Two phenylalanines (**FF**) in an **a**cidic **t**ract (FFAT)-motifs were originally described as having seven elements: an acidic flanking region followed by 6 residues (EFFDA–E). Such motifs are found in several **l**ipid **t**ransfer **p**rotein (LTP) families, and they interact with a protein on the cytosolic face of the ER called **v**esicle-associated membrane protein-**a**ssociated **p**rotein (VAP). Mutation of which causes ER stress and motor neuron disease, making it important to determine which proteins bind VAP. Among other proteins that bind VAP, some contain FFAT-like motifs that are missing one or more of the seven elements. Defining how much variation is tolerated in FFAT-like motifs is a preliminary step prior to the identification of the full range of VAP interactors.

**Results:**

We used a quantifiable in vivo system that measured ER targeting in a reporter yeast strain that over-expressed VAP to study the effect of substituting different elements of FFAT-like motifs in turn. By defining FFAT-like motifs more widely than before, we found them in novel proteins the functions of which had not previously been directly linked to the ER, including: two PKA anchoring proteins, AKAP220 and AKAP110; a family of plant LTPs; and the glycolipid LTP phosphatidylinositol-**f**our-phosphate **a**da**p**tor-**p**rotein-2 (FAPP-2).

**Conclusion:**

All of the seven essential elements of a FFAT motif tolerate variation, and weak targeting to the ER via VAP is still detected if two elements are substituted. In addition to the strong FFAT motifs already known, there are additional proteins with weaker FFAT-like motifs, which might be functionally important VAP interactors.

## Introduction

The function of intracellular proteins is largely determined by their precise targeting within cells, and recruitment of cytoplasmic proteins to membranes contributes a major part of organellar identity [1]. Peripheral membrane proteins make up a large proportion of overall membrane-associated protein [2], and often bind with a moderate affinity (micromolar to nanomolar dissociation constants) allowing regulatable and reversible recruitment. The membrane receptor can be a lipid or protein, and often a combination of more than one, allowing targeting by co-incidence detection [3]. Recruitment of cytoplasmic proteins to the endoplasmic reticulum (ER) has been demonstrated for components of ER membrane complexes that mediate (retro-) translocation [4], [5], and vesicle fission/fusion [6], [7].

While many proteins on the ER can recruit a small number of cytoplasmic binding partners, one pair of ER proteins recruits a wide range of partners: **v**esicle-associated membrane protein-**a**ssociated **p**rotein (VAP)-A and VAP-B are highly homologous ∼240 a.a. proteins conserved in all eukaryotes consisting of a single transmembrane helix anchoring a cytoplasmic domain [8], [9]. VAPs are important partly because dysfunction of VAP-B causes motor neuron disease [10], with one proposed mechanism being that lack of VAP-B causes ER stress [11], [12], [13]. Both VAPs bind with micromolar dissociation constants to FFAT motifs, standing for two phenylalanines (**FF**) in an **a**cidic **t**ract [14], [15], [16], motifs that occur in many different proteins (see below). However, it is not known which of the many interactions of VAP-B is related to motor neuron function, and importantly the full extent of VAP interactions has not been established.

The FFAT motif was precisely defined as ^1^EFFDA-E^7^ appearing in highly acidic, unstructured regions of multidomain cytoplasmic proteins [14], [15]. Each residue in the motif is involved in side-chain specific interactions with VAP [15]. There are six types of FFAT proteins, four of which interact with lipids: (1) the transcription factor Opi1p in yeast that binds phosphatidic acid [17]; (2) CERT, a lipid transfer protein (LTP) for ceramide by virtue of its StART domain [18]; (3) oxysterol binding protein-related proteins (ORPs), that have an LTP domain specific for sterols [19], [20]; (4) the phosphatidylinositol transfer protein subfamily related to rdgB in flies [21]. These optimal FFAT motifs have now all been shown to be functional in the context of full-length protein, leading to complex formation with VAP [17], [22], [23], [24], [25], [26]. However, the full-length proteins are still not clearly seen on the ER, with the sole exception of Opi1p. Instead, they often strongly target other sites (TGN, plasma membrane, late endosome). Despite this, FFAT motifs have been shown to be critical for function both in yeast and mammalian LTPs [14], [25], [26], [27], so it is thought that targeting to the ER occurs, but is hard to detect because the subdomains of ER involved form membrane contact sites with the TGN or plasma membrane [28], [29].

FFAT-like motifs differ from the canonical ^1^EFFDA-E^7^, but the degree of allowed difference has never been studied. FFAT-like motifs in ORPs show marginal difference from FFAT, and bind VAP tightly [23], [26], [30]. Although some VAP interactors, have no discernible motif like FFAT [31], [32], [33], [34], there are other examples of VAP interactors that have been suggested to contain FFAT-like motifs [35], [36], [37]. One is protrudin, an integral membrane protein that binds rab11 and migrates from the ER to endosomes, and which contains ^1^EFKDA-E^7^ [35], [38]. Another example is Glycolipid LTP (GLTP), a 209 a.a. protein that transfers glycolipids such as glucosylceramide (GlcCer) between membranes [39]. GLTP interacts directly with VAP, and a FFAT-like motif was identified in GLTP: ^32^PFFDC-G^38^, which has the ^2^FFD^4^ core [37]. Similarly, Orp3a, one of 12 ORPs in *Arabidopsis*, binds VAP via a small region within the ORP domain that has features of the FFAT core: ^2^WFD^4^ [36]. In protrudin, GLTP and Orp3a, mutations in the identified FFAT cores inhibited function, suggesting that FFAT-like motifs can vary considerably from ^1^EFFDA-E^7^ and still be physiologically relevant. This opens the question of how many proteins might be interacting with VAP, as sequences resembling the core ^2^FFD^4^ are extremely common (∼3,500 in humans), but only a small fraction of these are likely to be relevant.

Here, we started to define how much variation FFAT-like motifs can tolerate away from the optimal, canonical sequence. By studying increasingly divergent FFAT-like motifs, we have shown that each of seven elements of FFAT motifs (the six key residues plus the acidic flank) can be substituted, sometimes one element very suboptimally, or two elements both affected marginally. This approach led us to identify new proteins with FFAT-like motifs of varying strengths. Strong FFAT-like motifs in two of the 14 protein kinase A anchoring proteins (AKAPs) [40] tested positive for binding to VAP. A much weaker FFAT-like motif was found in the GLTP family member FAPP2. Sequences of LTP families particularly in plants, revealed new FFAT-like motifs. By comparing all examples of FFAT-like motifs, we developed an algorithm to score new sequences. This showed that while canonical FFATs make up the majority of strong interactors of VAP, there is a moderate number of new proteins with FFAT-like motifs that potentially target VAP. These merit further study as they may contribute to the role of VAP in ER stress.

## Results

### 1. Variation at every residue is found in FFAT-like motifs

The canonical FFAT motif ^1^EFFDA-E^7^ occurs in 29 eukaryotic cytoplasmic proteins, which are in 6 families: four lipid binding proteins (see above), (5) the worm homologue of Rab3GAP1, and (6) rabphilin-11 [14]. FFAT-like motifs with conservative substitution at a single position in ^1^EFFDA-E^7^ are found in homologues of ORPs, rdgB, Rab3GAP1 and rabphilin-11. These natural substitutions are: E^1^→D, F^2^→Y, F^3^→Y, D^4^→E, A^5^→C, E^7^→D, S or T [14]. The upstream flanking acids are conserved in all of these FFAT-like motifs. If all these single substitutions are tolerated, there are 127 variant combinations that might bind VAP. To gauge the importance of these variants, we identified where any of these 127 variations appeared in eukaryotic proteins. Only 30 of the possible 127 variant motifs were found, appearing 69 times. Twenty of these 69 hits are homologues of ORPs, rdgB, Rab3GAP1 and rabphilin-11. Although the effect of some of the variations (D^4^→E, A^5^→C) has not yet been reported, this conservation of FFAT-like motifs in protein families is a strong indicator that the key elements of the motif are functionally important. Apart from the twenty hits already known to us, there are 49 proteins with possible FFAT motifs containing one or more of the conservative variations (Table S1). These are analysed in greater detail in Section 4 (below).

**Figure 1:**
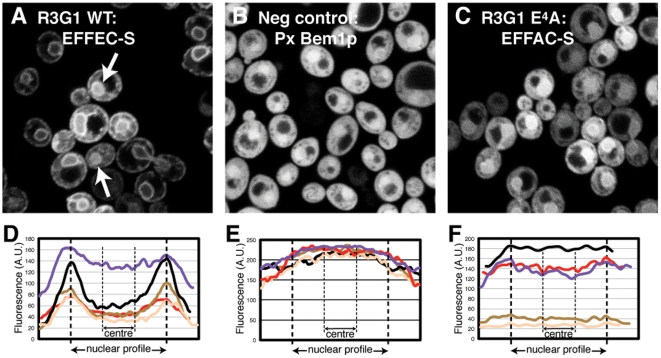
The FFAT-like motif of Rab3GAP1 targets VAP. (A) 26 amino acids including the FFAT-like motif ^1^EFFEC-S^7^ from Rab3GAP1 (see Table 1) were tagged with GFP and expressed in TLY251, which was grown in galactose to induce maximal yeast VAP (Scs2p) expression. Confocal sections through the plane of the nuclei showed the GFP construct interact with Scs2p, as fluorescence was found on the nuclear envelope (NE), in patches in the cell cortex, and in occasional strands in the cytoplasm, a pattern highly characteristic of the ER in yeast. Arrows indicate cells likely where the nucleus has been optically sectioned off the mid-line, so increasing central fluorescence and underestimating targeting. (B) As A, except GFP was tagged with the Px domain of Bem1p. This construct did not target membranes. (C) As A, except the FFAT-like sequence was altered at position 4: ^1^EFF**A**C-S^7^. ER targeting is barely detectable, but much weaker. (D/E/F) Fluorescence was measured across nuclear profiles from (A) (B) and (C) respectively, (examples shown in the insets, above). Line scans were re-plotted to normalise for different nuclear widths, and profiles from five nuclei are shown.

To test if the natural variation in these FFAT-like motifs affects ER targeting, we expressed the region of human Rab3GAP1 that contains the FFAT-like motif ^1^EFFEC-S^7^, with D^4^→E, A^5^→C and E^7^→S substitutions. The region surrounding the motif is highly acidic, and is predicted to form an unstructured loop (Table 1). We expressed this region in a GFP-tagged construct in a yeast strain that has inducible over-expression of Scs2p, the major yeast VAP homologue [14], [41]. The region of Rab3GAP1 targeted GFP to the ER, with fluorescence enriched at the nuclear envelope (NE), in patches in the periphery and in occasional strands crossing the cytoplasm (Figure 1A), a pattern highly characteristic of the ER in yeast. By comparison, GFP tagged with an irrelevant sequence failed to target any of these sites, with diffuse fluorescence (Figure 1B). As is characteristic for GFP itself, the negative control construct was slightly concentrated in the nucleus [42]. Similar diffuse targeting was seen with GFP-FFAT constructs when expressed in the same cells with repressed expression of VAP (data not shown), as found previously [14]. ER targeting by ^1^EFFEC-S^7^ from Rab3GAP1 is as strong as any FFAT motifs tested previously [14], [41], showing that FFAT-motifs tolerate E^4^, C^5^ and S^7^. This result is similar to the strong binding to VAP seen previously for ^1^EFYDA-S^7^ in human ORP1, varying at positions 3 and 7 [30]. Thus, multiple conservative substitutions are tolerated in FFAT-like motifs.

**Table 1:** Targeting strength ratios (TSRs) and number of suboptimal residues for all the FFAT-motifs studied.

Source	Core Sequence			Flanks		local±	H	TSR	#subopt
	amino	FFAT-like	carboxy	before	after				
Osh1-110+GAL	EDSDaD	EffDaEE	AA	23	72	-5		1.59	0
Osh1-110+DEX	EDSDaD	EffDaEE	AA	23	72	-5		0.90	0
Bem1p-Px	negative control			/	/	/		0.92	/
Rab3GAP1-wt	wSDSEE	EffEclS	DT	8	3	-4		1.76	0.5
Rab3GAP1-4A	wSDSEE	Eff**a**clS	DT	8	3	-4		1.12	1.5
Rab3GAP1-4Ae	w**E**D**E**EE	Eff**a**clS	DT	8	3	-6		1.08	1.5
AKAP220-wt	SDSEvS	EffDSfD	QF	5	9	-2	H	1.34	1
AKAP220–5E	SDSEvS	EffD**E**fD	QF	5	9	-2	H	0.91	2.5
AKAP220–5A	SDSEvS	EffD**a**fD	QF	5	9	-2	H	1.96	0.5
AKAP110-wt	lSSiEE	DflTaSE	HL	8	9	-2	H	1.17	2
AKAP110-4D	lSSiEE	Dfl**D**aSE	HL	8	9	-2	H	1.32	1.5
AKAP110-4A	lSSiEE	Dfl**a**aSE	HL	8	9	-2	H	0.99	2.5
Orp1c:Gm-wt	TDDDDN	affDTRD	IL	14	5	-4		1.36	1.5
Orp1c:Gm-AA	TDDDDN	a**aa**DTRD	IL	14	5	-4		0.89	4.5
Orp1c:Gm-1M	TDDDDN	**m**ffDTRD	IL	14	5	-4		1.34	1.5
Orp1c:Gm-1H	TDDDDN	**h**ffDTRD	IL	14	5	-4		1.11	1.5
Orp1c:Gm-1K	TDDDDN	**k**ffDTRD	IL	14	5	-4		1.01	2
Orp1c:Gm-1M4N	TDDDDN	**m**ff**n**TRD	IL	14	5	-4		1.02	2.5
Orp2a:At-wt	SEEDEp	SfhDTKE	FF	9	8	-4		0.94	2.5
Orp2a:At_**x2**	SEEDEp	SfhDTKE	FF	9	8	-4		1.33	(2.5)
Orp2a:At_**x2**-AA	SEEDEp	S**aa**DTKE	FF	9	8	-4		0.94	(4)
Orp2a:At_**x2**-1D	SEEDEp	**D**fhDTKE	FF	9	8	-4		1.36	(2)
Fapp2:Md-wt	EEEEvq	TffSamn	ED	8	9	-6		0.90	2
Fapp2:Md-1E	EEEEvq	**E**ffSamn	ED	8	9	-6		0.94	1.5
Fapp2:Md-4D	EEEEvq	Tff**D**amn	ED	8	9	-6		1.11	1.5
Fapp2:Md-1E4D	EEEEvq	**E**ff**D**amn	ED	8	9	-6		1.11	1
Fapp2:Hs_**x2-**wt	gKEvip	TffSTmn	TS	13	9	0		1.00	(4)
Fapp2:Hs_**x2**-1E4D	gKEvip	**E**ff**D**Tmn	TS	13	9	0		0.90	(3)
Fapp2:Hs_**x2-**EDA	gKEvip	**E**ff**DA**mn	TS	13	9	0		1.35	(2.5)
Fapp2:Hs_**x4-**wt	gKEvip	TffSTmn	TS	13	9	0		0.82	(4)
Fapp2:Hs_**x4**-4D	gKEvip	Tff**D**Tmn	TS	13	9	0		1.15	(3.5)
MAST205-wt	ESEDDT	SyfDTRS	ER	6	15	-4		0.90	2.5
Src-5A	lqaflE	DyfT**a**TE	PQ	10	7	-1	**H**	0.87	3
Ypt11p-wt	qghEqQ	EfhDTvE	EP	22	4	-2	H	0.86	3.5

The core FFAT-like motifs and immediate neighbours (6 amino-terminal and 2 carboxy-terminal) are shown for all sequences expressed in this study. Residues that might contribute to local charge (D, E, S, T, K or R) are in capitals, all others in lower case. Residues in bold indicate substitutions tested in this study. The number of other residues in the flanks of expressed constructs is also given. Full sequences and their precise origins are given in Table S5.

“local ±” is the sum of charges in eight residues flanking the motif (six before and two after): K/R = +1 and D/E =  –1.

“H” indicates that the region is predicted to be helical, **in bold** if known to be helical in structural studies (details in Table S2).

“TSR” is the “Targeting Strength Ratio” measured from nuclear profiles (see Materials & Methods), indicating the strength of NE targeting.

“# sub-opt” is the number of sub-optimal elements in each motif, determined according to the method set out in Table S2. Lower scores indicate a more optimal motif. Where the motif tested was multimerized the figures are in brackets, as they are not directly comparable.

We next studied the requirements for an acid at position 4, which we reported previously to be essential for ER localization [14]. Indeed, a Rab3GAP1 construct mutated E^4^→A (^1^EFF**A**C-S^7^) showed much reduced ER targeting (Figure 1C). To quantify the reduction in targeting, we used the least ambiguous aspect of ER targeting in confocal sections, which is the ring staining of the NE. Analyzing fluorescence across nuclear profiles on a cell by cell basis, ER targeting was calculated as the ratio of fluorescence on the NE to the central portion of the nucleus (Figures 1D/E/F). In making these analyses, we noted that the extent of NE targeting was lower in cells expressing more construct (Figure 1D). This shows that positive targeting was saturable, and indicates that FFAT proteins are capable of competing with each other.

The average ratio from multiple cells was calculated as **t**argeting **s**trength **r**atio (TSR, see Materials and Methods). To verify this approach we expressed a 110 residue region of Osh1p that contains one of the optimal FFAT motifs (Figure S1) [14]. Clear targeting of the FFAT-region of Osh1p was seen with high Scs2p, and the corresponding to TSR was 1.59. For the same construct in the same strain of yeast grown to repress Scs2p, the TSR was 0.90. A similar TSR was obtained with a negative control construct (Px domain of Bem1p) expressed in cells with high Scs2p, with TSR = 0.92 (Table 1). The reason that TSR was less than one for inactive sequences is likely to be that out of plane fluorescence makes the central portion of nuclei brighter than the perimeter. Now turning to the new FFAT-like motif, the Rab3GAP1 construct demonstrated stronger targeting than the Osh1p construct, with TSR = 1.76. Quantitation of targeting by the D^4^→A variant showed that it was not entirely negative: TSR = 1.12 (Table 1), significantly higher than control (Student's T-test: p<10^−8^). Substituting four upstream S→E did not increase NE targeting of the D^4^→A variant (Table 1), showing that targeting was limited by lack of D^4^, not overall acidity. A key finding from quantitation is that, while D^4^ or E^4^ is required for a strong interaction with VAP, a weak interaction can be obtained without it. If such weak interactions significantly increase the dwell time of cytoplasmic proteins on the ER, then potentially a very large number of FFAT-like motif variants could be active.

### 2. FFAT-like motifs in AKAPs – wider natural variation at positions 3, 4 and 5

**Figure 2:**
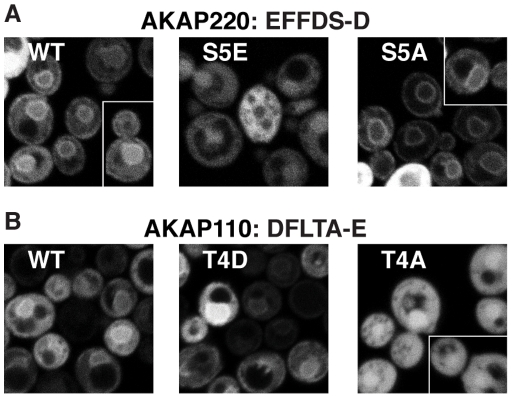
FFAT-like motifs in two AKAPs. (A) 29 amino acids from AKAP220 including the FFAT-like motif ^1^EFFDS-D^7^ (see Table 1) were expressed as in Figure 1A, together with variants at position 5: ^1^EFFD**E**-D^7^ and ^1^EFFD**A**-D^7^. The wild-type (WT) sequence showed moderate targeting, which was lost with E^5^ and enhanced by A^5^. (B) 32 amino acids from AKAP110 including the FFAT-like motif ^1^DFLTA-E^7^ (see Table 1) were expressed as (A), together with variants at position 4: ^1^ DFL**D**A-E^ 7^ and ^1^ DFL**A**A-E^ 7^. The wild-type (WT) sequence showed weak targeting, which was enhanced by D^4^ and lost with A^4^.

To begin to identify the far wider range of weaker FFAT-like motifs, we first searched for proteins with FFAT-like motifs as above, but now allowing for a new variation: A^5^ →S/T. Among 105 hits in total (14 in humans), one was AKAP220 (also called AKAP11), which has ^1^EFFDS-D^7^ in an acidic region near its amino-terminus. We expressed this region in cells, together with variants at S^5^→E and S^5^→A. ^1^EFFDS-D^7^ targeted the ER moderately, TSR = 1.3, showing that S^5^ is tolerated (Figure 2A). S^5^→E completely inhibited ER targeting (TSR = 0.9, Table 1), suggesting that phosphorylation at position 5 could be used to regulate FFAT-like motifs with S/T^5^. In contrast, S^5^→A enhanced targeting, producing the highest TSR of all motifs in this study (TSR = 1.96, Figure 2A, Table 1).

Examination of the rest of the amino-terminus of AKAP220 revealed a second FFAT-like motif nearby: ^1^DFVTA-E^7^. Not only are both motifs conserved in AKAP220 in other species (*e.g.* fish), but the second motif is conserved in the protein most closely related to AKAP220: AKAP110 (also called AKAP3, FSP95, fibrousheathin-1, SKIP, and SPKAP) has ^1^DFLTA-E^7^. These motifs are interesting as they have two substitutions: F/Y^3^ → non-aromatic hydrophobic residues and D/E^4^ → T. In yeast, the region from AKAP110 targeted VAP weakly (TSR = 1.2, Figure 2B). We tested if phosphorylation of S^4^ or T^4^ is one mechanism to regulate VAP binding by this FFAT-like motif, and found that the T^4^D variant targeted significantly better than the original sequence (TSR = 1.3), while the T^4^A variant largely failed to target (TSR = 1.0, Figure 2B, Table 1). Therefore, while T^4^ is tolerated, pseudo-phosphorylation of T^4^ increases ER targeting. These results show that two AKAPs in humans might be recruited to VAP, where they could regulate cAMP signalling on the ER [43], and also the results suggest a much broader molecular definition of FFAT motifs should be considered.

### 3. FFAT-like sequences in plant LTPs with natural variation in positions 1, 3 and 5

**Figure 3:**
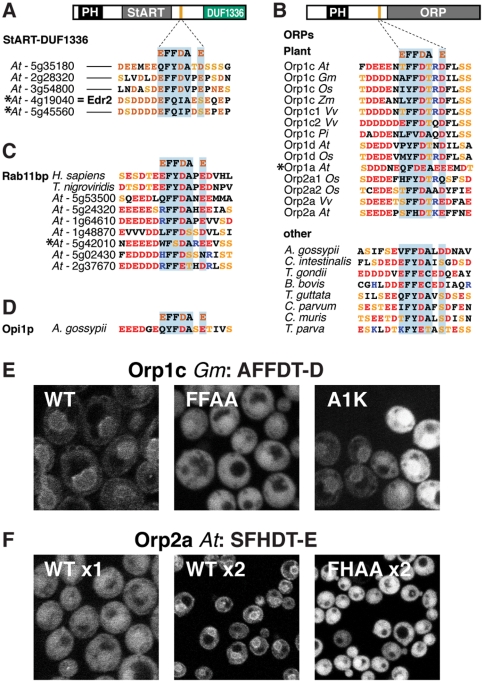
FFAT-like motifs in plant ORPs. (A–D) Aligned FFAT-like motifs from (A) the subfamily of StART proteins related to Edr2 in *Arabidopsis thaliana* (*At*); as shown in the diagram, this region is between the StART domains and DUF1336, which are domains of unknown function structurally related to galectins. (B) ORPs. FFAT-like motifs, which occur upstream of ORP domains. Top: in *At* and homologues in other plants: *Gm – Glycine max; Os – Oryza sativa; Zm – Zea mays; Vv – Vitis vinifera; Pi – Petunia integrifolia*; bottom: in other species, as listed. (C) homologues of rabphilin-11from human, fish (one each) and *At* (x7). These motifs are at the extreme amino-termini. (D) Opi1p in the fungus *Ashbya gossypii*. (E) 34 amino acids from Orp1c (*Gm*) including the FFAT-like motif ^1^AFFDT-D^7^ were expressed as in Figure 1A, together with variants ^1^A**AA**DT-D^7^ and ^1^ **K**FFDT-D^ 7^. The wild-type (WT) sequence showed moderate targeting, which was lost with ^2^AA^3^ and reduced with K^1^. (F) 32 amino acids from Orp2a (*At*) including the FFAT-like motif ^1^SFHDT-E^7^ were expressed as in (E) (left hand panel). We also expressed the dimer of this motif, and a dimer of a mutant: ^1^S**AA**DT-E^7^ (middle and right panels, images are at 2-fold lower magnification. The wild-type (WT) monomer failed to target, but moderate targeting was obtained upon dimerization, which was lost with ^2^AA^3^.

Among the 15 human LTPs that contain StART domains only CERT has a FFAT motif [44]. StART domain proteins are amplified in plants (35 in *Arabidopsis*) [44], but none contain FFAT motifs. Using the wider definitions from our work on AKAPs, we noted that three of the sub-group of five plant StART proteins including Enhanced Disease Resistance (Edr)-2 have FFAT-like motifs with single substitutions E^1^→Q or A^5^→V (Figure 3A). Also in plants, our original study had suggested a possible FFAT-like motif in a conserved acidic region in *A. thaliana* Orp1c. We now realise that the motif we predicted before (^1^EEFDE-E^7^) is likely to fail on account of both E^2^ and E^5^. However, directly adjacent to this is the sequence ^1^TFFDT-D^7^, which was not originally obvious as a FFAT-like motif, but includes the ^2^FFD^4^ core. Among other *A. thaliana* ORPs, Orp1d is similar to Orp1c with ^1^PYFDT-D^7^, Orps2a/2b have ^1^SFHDT-E^7^, and Orp1a has the acidic tract but the motif is highly altered to ^1^QFDEA-E^7^ (Figure 3B). FFAT-like motifs at this site in ORPs of other plants naturally vary at position 1 to any of AEHILMPST (Figure 3B). Similar variation is also seen in diverse ORPs in other species (Figure 3B), in plant homologues of the FFAT protein rabphilin-11, a WD repeat protein implicated in vesicular trafficking (Figure 3C) [45], [46], and in a fungal homologue of Opi1p (Figure 3D), with position 1 any of EHIKLQRTVW. These natural variants imply that any residue is tolerated at position 1, particularly in plants.

We examined putative plant FFAT-like motifs to test the significance of variation at position 1. First we expressed ^1^AFFDT-D^7^ from Orp1c in *Glycine max* (soybean). This targeted moderately well (Figure 3E, TSR = 1.36, Table 1). Mutation of the key residues ^2^FF^3^ reduced targeting to background levels (Figure 3E, Table 1), demonstrating the expected specificity of interaction. We varied position 1, and found that a hydrophobic residue (^1^ **M**FFDT-D^7^) had no effect on targeting, while H^1^ reduced targeting (Table 1). Introduction of K^1^, the polar opposite of the typical acid, reduced targeting to a barely detectable level (TSR = 1.01, Figure 3E, Table 1). Although targeting was above background (p = 5×10^−4^), K^1^ was quite inhibitory (Table 1). This shows that a highly unfavourable substitution at position 1 can inhibit binding considerably, even though this position is the least constrained of the seven elements of a FFAT motif residue [15].

Given that a F^3^H substitution is found in a *C. elegans* homologue of oxysterol binding protein [14], we decided to test the FFAT-like motif in *A. thaliana* Orp2a and Orp2b: ^1^SFHDT-E^7^ with highly acidic flanks. When expressed in cells, this showed no ER targeting (Figure 3E, Table 1, TSR = 0.94, not significantly above background). To detect any weak targeting that might exist for this motif, we expressed a construct containing two of the motifs as a tandem dimer, similar to the approach used to enhance weak targeting of PH and FYVE domains [47], [48]. Dimerization is used here to increase avidity, and this reproduces aspects of the *in vivo* situation, since VAP in many species (but not yeast) dimerizes [49]. Dimeric ^1^SFHDT-E^7^ showed moderate NE targeting (Figure 3E, Table 1). The specificity of this was shown by loss of targeting with dimeric ^1^S**AA**DT-E^7^ (Figure 3E, Table 1), which indicates that the binding does not result solely from the increased electrostatic interaction of the double motif (overall charge −20). The functional effects of clustering of Scs2p by the dimeric motif are unknown, but the clustering is unlikely to be critical for binding, given that strong interactions occur with monomeric FFAT motifs. Since ^1^SFHDT-E^7^ could be phosphorylated at position 1 to mimic the canonical D/E, we tested the effect of a S^1^D substitution in dimers. This did not enhance targeting over wild-type (Table 1). This is consistent with S^1^ already being phosphorylated in yeast; this residue is a predicted Casein Kinase II site. Thus, 4 of the 12 plant ORPs have FFAT-like motifs, which are active even with three substitutions: S^1^, H^3^ and T^5^. Again this suggests that many substitutions in FFAT motifs are tolerated if other residues are optimal.

### 4. Assessing the extent of FFAT-like sequences in all human proteins

Our findings above show that FFAT-like motifs can tolerate multiple substitutions. In another study, the endosomal regulator protrudin (also called ZFY27) was found to contain a FFAT-like motif ^1^EFKDA-E^7^, which binds to VAP, and is inhibited by a D^4^A mutation, despite the F^3^→K substitution [35].This shows that FFAT-like motifs also tolerate single extreme substitutions, Therefore, we then used our knowledge of substitutions at each position in the motif to generate a simple tool to assess how optimal any FFAT-like motif is overall (Table S2A, see Materials and Methods). This tool was used to determine the number of suboptimal elements in any putative motif, with the strongest FFAT motifs scoring zero, and very poor motifs scoring up to seven. To check the tool, we first scored the FFAT-like motifs that we had expressed in yeast (Table 1, final column). This showed that FFAT-like motifs that target at barely discernible levels tend to score 2 suboptimal elements, but that lack of ≥2.5–3 elements inhibited targeting. Next, we applied these requirements to score FFAT-like motifs in all human proteins. We limited these searches to human proteins since there would have been too many sequences to handle (>20,000) if the entire database was queried. Using a cut-off of ≤2 suboptimal elements, 76 human sequences were found, including all of the 14 human FFAT motifs identified originally [14], [15], and 62 new FFAT-like motifs. Increasing the cut-off even marginally would identify many more putative FFAT-like motifs (Table S2B).

Pooling the 62 new human FFAT-like motifs with 2 or less elements missing together with the 49 new motifs from all species containing one of the 127 simple variant FFAT-like motifs (Table S1), and discounting repeats, we arrived at a list of 79 proteins with new possible FFAT-like motifs (Table S3). Four criteria were then applied, where likely interactors have to meet all four: (A) they are cytoplasmic; (B) they do not definitely form helices in known crystal structures of homologous domains, as helical arrangement of the motif will not allow it to extend across its binding site in VAP [15], [30]; (C) the critical residues of the FFAT-like motifs are conserved among orthologues more than the neighbouring residues; and (D) the motifs have 2 or fewer elements missing, this being the cut-off we determined for detectable targeting (Table 1). This criterion had to be applied to the 49 motifs identified in Table S1, because firstly some of the natural variations appear suboptimal, in particular F^2^→Y, but also F^3^→Y and E^7^→S/T, and secondly their flanks were not all acidic. For example, EFYDA-S in Rad2p (yeast) is preceded by HEKNYV. Where structure was predicted, but not known from direct study, predictions were not used to exclude a motif. The four criteria were met by AKAP220, AKAP110, protrudin and 18 proteins previously not known to contain FFAT-like motifs (Table S3). 58 remaining proteins with FFAT-like motifs failed to meet one or more criterion (Table S3).

The 18 motifs that meet all our criteria are good candidates for interactors of VAP. Among these, only three proteins contain a motif very close to the optimum, (*i.e.* missing one element or less): Vps13C, SLC22A15/Flipt and rhophilin-1 (Table S3). Vps13C is one of four human homologues of yeast Vps13p, which is a cytoplasmic protein >3000 a.a. implicated in membrane trafficking. Vps13C has a FFAT-like sequence in a predicted unstructured loop, and a very similar motif is present in the same loop of Vps13A (also called chorein), the closest homologue of Vps13C [50]. Similar FFAT-like motifs are found in most vertebrate Vps13C orthologues, in distantly related organisms such as slime mold and many fungi. SLC22A15/Flipt, (Solute Carrier (SLC)-22 member 15), is a 12 transmembrane helix protein. It has a FFAT-like motif in the 60 a.a. cytoplasmic domain at its extreme carboxy-terminus, and the motif in Flipt is conserved in all its orthologues, but not found in the >20 other SLC22 family members. Rhophilin-1 and its orthologues interact with Rho-GTPase, and contain a strong FFAT-like motif in a region predicted to be unstructured. Since the motif in AKAP220 is able to target VAP, we predict that the motifs in Vps13C, Flipt and rhophilin-1 can also bind VAP, and that these proteins are at least partially targeted to the ER. Interestingly, rhophilin-1 also binds ropporin, which itself interacts with AKAP110 [51], [52], suggesting that two components of the same complex can both interact with VAP.

Another way to identify proteins with FFAT-like motifs is to look for motifs among VAP interactors identified in high-throughput experiments. The BIOGRID database lists 13 interactors for VAPB (Figure S2A), two of which have FFAT-like motifs. One is USP20 [53], one of the large family of deubiquitinases. USP20 has a long unstructured loop with a slightly sub-optimal FFAT-like motif (^282^EFLSC-S^288^) that is reasonably conserved in all orthologues (Figure S2B). A weaker FFAT-like motif is also found in USP33, the closest homologue of USP20 (Figure S2B). The second VAP interactor with a FFAT-like motif is the Regulator of Microtubule Dynamics-3 (RMD3, also called PTPIP51 or FAM82A2) [54], an integral membrane protein on the outer face of mitochondria. While this paper was in revision, RMD3 was shown to bind VAPB across ER-mitochondrial contact sites to modulate calcium traffic from ER to mitochondria [55]. Although not commented on in that paper, we noted that the minimal portion of human RMD3 required for VAP binding (residues 84–175) contains not one but two FFAT-like motifs, one of which (^157^VYFTASS^163^) is highly conserved in all RMD3 orthologues. One of these contains the strong motif ^1^EFYEA-Q^7^ (Figure S2C). RMD2, a close homologue of RMD3, also contains a relatively active FFAT-motif, so RMD2 (but not RMD1) may be participating in the same functions as RMD3. Although we have not tested the FFAT-like motifs in USP20 and RMD3, our analysis suggests a possible mechanism to regulate the function both of USP20 in receptor recycling [56] and of RMD3 in apoptosis [55]: by phosphorylation of their FFAT-like motifs.

### 5. FFAT-like sequences in short and long isoforms of GLTP indicate three possible locations for FFAT-like motifs in GLTP and FAPP2

**Figure 4:**
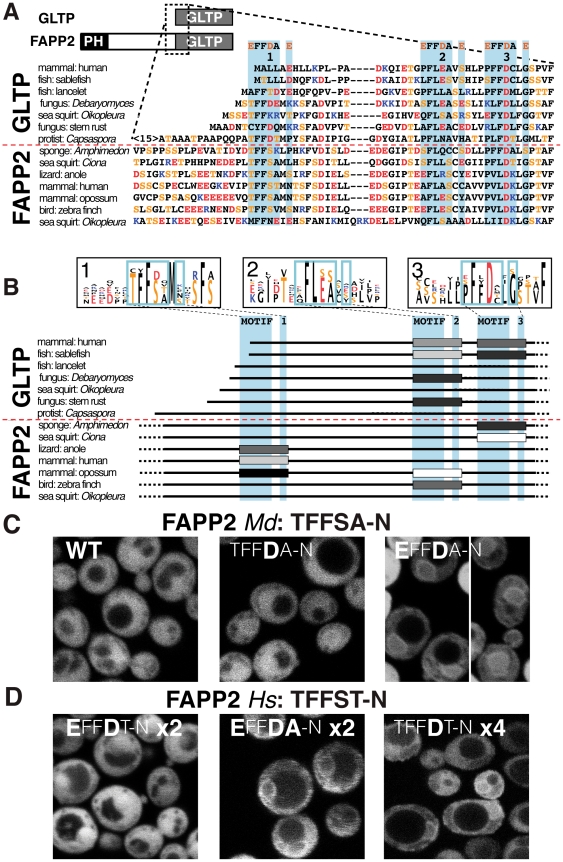
FAPP2 in mammals has a weak FFAT motif. (A) An alignment of ≤60 a.a. from the region at the amino-terminus of 7 divergent GLTP sequences and the related region near the middle of 7 divergent FAPP2 sequences (see diagram, top). Three motifs are shown where the core ^2^FFD^4^ is partly conserved. (B) Diagram of the aligned sequences from (A), where the FFAT-like motifs are shown as logos, and those individual motifs that meet minimal criteria are shown as boxes, where darker shading indicates closer resemblance to the optimal motif (see Tables S2 and S3). The best FFAT-like motif lies at different positions in different species. The most amino-terminal motif in human FAPP2 is quite weak. (C) 32 amino acids from FAPP2 in the opossum *M. domestica* including the FFAT-like motif ^1^TFFSA-N^7^ were expressed as in Figure 1A, together with variants ^1^ TFF**D**A-N^ 7^ and ^1^ **E**FF**D**A-N^7^. While the wild-type (WT) sequence did not target, the variants with D^4^ targeted weakly. (D) The dimer of 37 amino acids including the FFAT-like motif from human FAPP2 with two substitutions ^1^ **E**FF**D**T-N^7^ (see Table 1) were expressed as in (C), together with a dimer carrying a third change ^1^ **E**FF**DA**-N^7^, and a tetramer of ^1^TFF**D**T-N^7^. While the dimer with E^1^/D^4^/T^5^ failed to target, the E^1^/D^4^/A^5^ version targeted moderately, and the tetramer with D^4^ targeted weakly.

Compared to CERT and ORPs with canonical FFAT motifs, GLTPs do not obviously contain a FFAT motif, even though they are implicated in similar pathways of intracellular traffic [57], [58]. Human GLTP binds VAP via ^1^PFFDC-G^7^ (residues 32–38), a motif that contains the FFAT core ^2^FFD^4^ [37], but is missing three of the key FFAT elements (Table S4). Examining sequences similar to ^1^PFFDC-G^7^ in the amino-terminus of diverse GLTP sequences, we found a previously unreported FFAT-like motif just upstream (Figure 4A). In some GLTPs (for example, from the fungus *Debaryomyces*), this motif is closer to optimal than the downstream one (Table S4). This implies that GLTPs have multiple weak FFAT-like motifs that may act in concert.

GLTPs have short and long isoforms (Figure 4A), the former consisting of the LTP domain alone, the latter extended to include a PH domain that targets the TGN, hence called phosphatidylinositol-**f**our-phosphate **a**da**p**tor-**p**rotein-2 (FAPP2) [59]. This same pattern of short and long isoforms is also seen with CERT and ORPs [44], [60]. FAPP2 functions to traffic GlcCer, which is made in the Golgi [57], [59]. However, it is controversial where GlcCer is delivered by FAPP2, possibilities including the TGN [57], or the ER [58]. We therefore asked if FAPP2 shares another property of CERT and ORPs, namely does it contain a FFAT-like motif to target the ER? An alignment of diverse FAPP2 and GLTP sequences showed that some FAPP2s share the FFAT-like motifs found in GLTP. Furthermore, some FAPP2s have another previously unreported FFAT-like motif upstream of the other two (Figure 4A). We scored the three potential motifs in this region for how many elements are optimal. Significantly, some of the new FFAT-like motifs in FAPP2 score similarly to the GLTP motif that is functionally relevant (Table S4) [37], so they might also contribute to function.

To test if FAPP2 can bind VAP, we expressed the most optimal mammalian FAPP2 motif, ^1^TFFSA-N^7^, which occurs in marsupials such as opossum (Figure 4B). This did not target the ER (Figure 4C), neither did a ^1^ **E**FFSA-N^7^ variant that mimics phosphorylation at T^1^ (data not shown). However, pseudo-phosphorylation of S^4^ with ^1^TFF**D**A-N^7^ did produce a weak interaction with VAP, as did the double substitution ^1^ **E**FF**D**A-N^7^ (Figure 4C, Table 1). This shows that if FAPP2 in species such as opossum was phosphorylated at S^4^ of this motif (S317), the whole protein might be recruited to the ER. The FFAT-like motif of FAPP2 in humans (^1^TFFST-N^7^) is less optimal than in opossum in 2 elements: fewer flanking acids, and A^5^→T (Figure 4A). Since T^5^ can be tolerated (Fig. 3), we investigated ER targeting of this region. Both the wild-type sequence and a variant with two pseudo-phosphorylations (^1^ **E**FF**D**T-N^7^) failed to target VAP on the ER (data not shown). Also a dimeric version of the latter motif showed no ER targeting, and only after adding in a third change T^5^→A did we detect ER targeting (Figure 4D, Table 1).

The finding that the dimeric human FAPP2 sequence only targets when it has A^5^ suggests that it is far from optimal. Nevertheless, because FAPP2 itself is normally dimeric [61], together with VAP being dimeric [49], it is possible that a very weak FAPP2-VAP interaction would be magnified by avidity effects to become functionally significant. To attempt to model these avidity effects, we expressed constructs with four copies in series of the FAPP2 motif. Even though there is no evidence that FAPP2 is tetrameric, and the tetramerization does not reproduce the correct three-dimensional distribution, this will increase avidity, as in the use of 13x-myc epitope tags [62]. Our result with a tetramer of wild-type human FAPP2 motif was again that it did not target (Table 1). However, tetrameric ^1^TFF**D**T-N^7^ now did weakly target the ER (Figure 4D, Table 1). Therefore, pseudo-phosphorylation of S^4^ in the FFAT-like motif of human FAPP2 does produce a weak interaction with VAP. This modification could be the basis for a regulated interaction of FAPP2 with VAP that enhances GlcCer traffic to the ER.

## Discussion

Here, by testing a range of FFAT-like motifs, we have defined which substitutions in this very small motif are neutral to ER targeting, and which are sub-optimal. Even with sub-optimal substitutions, strong targeting can occur if five or six of the seven elements (6 key residues + acidic tract) are optimal. We still do not know the full extent of allowed variation, but the highly detrimental substitution F^3^→K is allowed in protrudin [35], showing that any single detrimental might be allowed. The approach we used here was *in vivo* expression of regions of proteins. Compared to an alternate approach of *in vitro* binding by peptides to VAP, this has the advantage that post-translational modifications such as phosphorylation can occur. However, our experiments fell short of providing dissociation constants, which would be useful. Such direct binding was reported for optimal FFAT and seven variant peptides, where single substitutions reduced the affinity of binding between 2 and 15-fold [15]. Our experiments suggest other peptides that might be tested *in vitro*. Also, we can now suggest which full length proteins might be studied for direct binding to VAP *in vivo* (Table S3). Prior identification of targets is helpful because it has proved difficult to demonstrate interactions of VAP with full-length proteins, even when they have strong FFAT motifs that are known to be important for protein function (for example: ORP9L [23], [27]).

A major assumption that underlies our work is that the residues on VAP that are involved in FFAT binding are conserved, so allowing conclusions from binding to VAP in yeast to be applied to proteins in all species. 11 residues have been identified in VAP that contact FFAT motifs [15], and these are highly conserved in all eukaryotes (Figure S3), so we have assumed that the requirements for FFAT motifs are the same in all proteins, though this has not been studied directly. Another issue to be considered is whether VAP is restricted to the ER. There are reports of VAP outside the ER [63], [64], but this has not been reproduced more widely. Instead VAP has been shown to move within the ER to membrane contact sites, with the TGN, the plasma membrane and mitochondria [20], [25], [55], [65], and the VAP interactors are strong candidates for proteins that bridge such contact sites [28]. Thus, we hypothesize that all proteins containing functional FFAT-like motifs will to some extent be components of the ER, including the inner NE, since components of the nuclear transcriptional machinery can bind VAP [66]. A point that might counteract this is the possibility that proteins with FFAT-like motifs compete for a limited number of VAP molecules. In yeast, where the number of all proteins has been estimated, the copy number of Scs2p only just exceeds that of all 4 proteins with strong FFAT motifs [67], but we implicated an increasing number of proteins as having FFAT-like motifs rises, it will become more important to determine the extent of competition.

Some of the substitutions in FFAT-like motifs we have studied allow direct phosphorylation that can modulate affinity for VAP. Regarding residue 5 of the motif, our results with AKAP220 (Figure 2) are informative here. A^5^ normally fits in a hydrophobic pocket [15], [30]. E^5^ completely inhibited ER targeting, suggesting that a negative charge is not tolerated in the pocket. This leaves the question of why S^5^ targets significantly worse than A^5^. This is unlikely to be caused by steric hindrance, because C^5^, which is larger than S^5^, is well tolerated (Figure 1). Therefore, the weaker targeting of S^5^ compared to A^5^ suggests that S^5^ might be partially phosphorylated in yeast, to mimic E^5^. While there is no experimental evidence for this phosphorylation, the upstream residues partly match the consensus of ATM kinases, and although ATM phospho-sites usually have Q at position +1, this is not essential [68]. Therefore, phosphorylation of S^5^ or T^5^ remains a possible (but untested) way in which FFAT-like motifs might be negatively regulated. In contrast to inhibitory phosphorylation at position 5, phosphorylation at positions 1, 4 and 7 may activate FFAT-like motifs, in particular phosphorylation of core residue 4, as in FAPP2 (S^4^) and AKAP110 (T^4^) (Table 1). In our assays, the FFAT-like motif in vertebrate FAPP2 only functions if it is activated by pseudo-phosphorylation. Presumably tyrosine phosphorylation can also regulate FFATs, which might explain why many proteins have conserved Y^3^ in place of the canonical F^3^.

The FFAT-VAP interaction occurs in two stages: an initial electrostatic interaction between the acidic tract and the electropositive face of VAP is followed by a “fly casting” step, with the core of the motif then binding by a lock-and-key interaction with specific residues in VAP [15]. We have now found several FFAT-like motifs which have second, weaker motifs nearby in the sequence: in *Arabidopsis* Orp1d has ^1^DFYSV-S^7^ 30 residues before ^1^PYFDT-D^7^ and Orp2a/2b have ^1^EFEDV-E^7^ upstream of ^1^SFHDT-E^7^, and Osh1p in yeast has ^1^EFFDK-N^7^ 120aa before ^1^EFFDA-E^7^. Likewise some GLTP and FAPP2 homologues have two FFAT-like motifs, though none have three. According to the “fly casting” model, a large acidic patch would enhance the initial, relatively nonspecific electrostatic interaction, allowing a suboptimal motif to bind. This enhancement might explain how substitutions in key residues are tolerated: K^3^ and I^4^ are only found in human protrudin and the StART protein Edr-2 respectively, while Y^2^ is found in human ORP4 but is otherwise rare (Table 1 and Figure 3). In each case, these FFAT motifs are preceded by at least seven closely packed D/Es. AKAP220 is unique in having two strong FFAT-like motifs. The reasons for this are obscure, but might be because the two motifs can be either inhibited (^1^EFFDS-D^7^) or activated (^1^DFVTA-E^7^.) by S/T phosphorylation.

The significance of FAPP2 is that it is the GLTP that transports GlcCer, and one proposed route of traffic is from the TGN to the ER [58]. While FAPP2 in some species has a strong enough motif to plausibly bind VAP, in mammals the FFAT-like motif is less well preserved than in other animals, so it could only be a very weak interactor of VAP, and it is unlikely that the FAPP2-VAP interaction has been positively selected during mammalian evolution. The ER-related functions of other novel proteins with FFAT-like motifs are not known. Rab3GAP1 was originally identified as a GTP-ase activating protein (GAP) specific to Rab3 [69], but recent studies that included all Rab GAPs did not replicate this finding [70], so the true function of Rab3GAP1 is open for speculation. Intracellular localizations described for AKAP220 and AKAP110 do not include the ER [71], [72], but there are roles for PKA/AKAP at that site [43], and Rab32 is another AKAP, which has been localised to part of the ER [73]. cAMP signalling occurs in highly localized zones <1 µm in diameter [74], so precise targeting of AKAPs is a critical aspect of cell function.

How many other proteins bind VAP? Based on our results, which focussed mainly on human proteins, we tentatively suggest a list of 18 new proteins with possible FFAT-like motifs (Table S3). The most likely candidates among this list are: Vps13A/C, rhophilin-1 and Flipt/SLC22A15, all of which contain only naturally occurring substitutions (Table S1). One criterion we applied was to exclude motifs occurring in helices, which is based on findings that FFAT motifs bind VAP in an extended sheet [15], [30]. However, the motif in GLTP is in a 3–10 helix [75], [76], and this motif along with that in Orp3a in *Arabidopsis* both fail to meet our criteria for defining motifs (Table S3, bottom), so it is not clear whether the structural criterion is helpful. Applying our criteria more loosely, a very long list of even weaker FFAT-like motifs could be generated (Table S2B). We tested three hits just below the cut-off, *i.e.* with more than two suboptimal FFAT elements (Table 1): microtubule-associated serine/threonine kinase (MAST205), c-Src, and the ER-targeted Rab-GTPase in yeast Ypt11p. None of these targeted the ER (data not shown, Table 1), which lends support to the cut-off for detectable binding of monomeric proteins being two suboptimal FFAT elements. However, we realise that we only partially understand what constitutes an optimal FFAT motif. For example, it is not clear why the FFAT-like sequence from FAPP2 in opossum failed to target as monomers even after key residues were “corrected” by substitution.

Overall, we find that the very short FFAT motif tolerates considerable substitution, so that physiologically significant FFAT-like motifs are hard to detect, making it hard to fully map the VAP interactome. The broad spectrum and diverse intracellular localizations of proteins with FFAT-like motifs suggest that VAP-A and VAP-B are major platforms for integrating many novel inputs into the ER. This shows that we still cannot accurately predict the physiological significance of the loss of VAP, for example in motor neuron disease.

## Methods

Constructs containing the sequences shown in Table S5 were cloned by ligating oligonucleotides in a plasmid based on pRS406 expressing GFP-myc from the PHO5 promoter [41]. To express dimers, plasmid fragments were ligated together to produce GFP-myc-motif1-myc-motif2. For tetramers, fragments of dimers were ligated to further duplicate the insert. Plasmids were integrated into strain TLY251, which has the *GAL1/10* promoter upstream of the coding sequence of *SCS2* [14]. Colonies were grown overnight in 2% galactose, (except for the Osh1p-FFAT experiment, where a parallel culture contained 2% dextrose as carbon source), diluted back in the same medium for 6–8 hours to reach log phase, and examined by live cell confocal microscopy. Strength of targeting was analyzed by drawing a line 15 pixels wide across individual nuclear profiles in ImageJ, to produce a line scan plot (five such plots shown in Figure 1D/E/F). From each plot the ratio was calculated of mean fluorescence at the nuclear rims (peak pixel plus one on either side) compared to the average fluorescence across the central third of the nucleus (equivalent to “central region” in Figure 1D/E/F). The **t**argeting **s**trength **r**atio (TSR) was calculated from the average of these ratios ≥20 cells.

To find FFAT-like motifs with the natural variations E^1^→D, F^2^→Y, F^3^→Y, D^4^→E, A^5^→C, E^7^→D, S or T, we screened the entire UniProtKB/Swiss-Prot database of eukaryotic sequences using the ScanProsite tool (prosite.expasy.org) using the pattern [DE]-X(0,5)-[DE]-[FY]-[FY]-[DEST]-[ACST]-X-[DEST], which ensures at least one acid among the 6 residues upstream of the desired 128 variant motifs. This generated 100 hits (Table S1). For a screen of motifs using a wider definition based on our findings, the UniProtKB/Swiss-Prot database of human sequences was scanned using, starting with the pattern [DE]-X(0,5)-X-[FY]-[FYCILMVWH]-[DEST]-[ACST]-X-[DESTGNQ], which allows for extra variation at positions 1, 3, 4 and 7, and produced >3,000 hits. Other patterns were also used to include more extreme variants at individual residues. All potential motifs were then compared to the optimal FFAT to determine how many of seven elements (6 residues + acidic flank) they lack. Scoring was based on our analysis of the combination of (1) the effects on targeting of single mutations, and (2) the natural variation seen in the ORP family. Firstly, we analysed how to weight the seven elements. Variation in residues 1/2/3/4/7 had seemingly similar impact on ER targeting (Table 1 and Figure 3), so we weighted these elements equally (1 arbitrary unit). By comparison, variation at position 5 between AC and ST was less significant, while the variation in charge in the upstream region appeared to be more significant, so these two elements were weighted at 0.5 and 1.5 units respectively. Secondly, we determined which residues are partially acceptable, allocating intermediate penalties compared to maximum penalty at that residue (Table S2A). In addition, we estimated penalties for highly divergent residues (Table S2A). The same scoring system (higher score = worse resemblance to FFAT) was applied to all the FFAT-like motifs we expressed in yeast (Table 1, final column) and to motifs obtained from alignments (Table S4).

Structural predictions were made by PSIPRED 3.0 [77], coiled-coil prediction was made by COILS [78], and phospho-site prediction was made by Scansite [79]. Alignments with known structures were made with HHpred [80]. VAPs were aligned with MUSCLE. To determine evolutionary conservation of FFAT-like motifs (Table S3), each protein was submitted to BLAST at NCBI to identify homologues from the non-redundant database.

## Supporting Information

Figure S1 **Relationship between FFAT motif targeting the ER and expression of Scs2p.** The FFAT-containing region of Osh1p (residues 687–796) was expressed from pTL377 [14] in TLY251, which was grown either in galactose (+GAL) to induce maximal expression of yeast VAP (Scs2p), or in dextrose (+DEX) to repress expression. As in Figure 1, fluorescence was measured across nuclear profiles to produce targeting strength ratios, see Table 1.
(TIF)

Figure S2 **Possible additional FFAT-like motifs found by data mining.** A. Interactors of VAPB currently listed at BIOGRID (http://thebiogrid.org/). The bottom 8 were found in targeted studies of VAP-SNARE interactions, and bind via the coiled coil and/or transmembrane domain. The top 5 were found in high-throughput studies. Of these, one has a known FFAT (ORP9), and two others have FFAT-like motifs that might mediate the interaction with VAP: USP20 and RMD3 (see parts B and C). **References:** 1. Sowa *et.al.* (2009) Cell 138: 389–403; 2. Gong *et al.* (2006) PNAS 103: 6154–6159; 3. Hutchins *et al.* (2010) Science 328: 593–599; 4. Nishimura *et al.* (1999) BBRC 254: 21–26; 5. Li *et al.* (2003) JBC 278: 19791–19797. B. A FFAT-like motif in USP20 is well conserved. An unstructured loop in human USP20 contains a FFAT-like motif that is marginally suboptimal (has more than 2 suboptimal elements in most but not all species), but is well conserved not only in all vertebrates but also in primitive metazoa such as *Trichoplax*. A version of the same motif appears in a more restricted group of USP33 homologues: vertebrates excluding birds. C. Molecular mechanism underlying the RMD3-VAP interaction. RMD3 (also called PTPIP51 or FAM82A2) on the outer mitochondrial membrane binds VAP on the ER across the ER-mitochondrial contact site [55]. The molecular basis for the interaction has not been studied, but the minimal VAP-binding sequence in human RMD3 (light blue) contains two adjacent FFAT-like motifs, both of which are sub-optimal, so would not appear in our list of most likely interactors (Table S3). The motif is well conserved in RMD3 orthologues, and is more optimal in sea squirt (*Ciona intestinalis*) and sea anemone (*Nematostella vectensis*). The latter motif meets all our criteria for a strong FFAT-like motif (see Table S3, second section). The motif is weakest in fish, which have a highly acidic tract in the downstream region (asterisk). In addition, RMD2 in some species (but not man) contains a FFAT-motif. Other domains in RMD3 and VAP are: yellow = trans-membrane domains, grey = tetratricopeptide repeat domain, red = MSP domain, green = coiled coil.
(TIF)

Figure S3 **Residues in VAP that bind FFAT are conserved well throughout all eukaryotes.** The amino-terminal MSP domains of all VAPs in the genomes of diverse model organisms (humans-*Hs*, flies-*Dm*, worms-*Ce,* plants-*A*t, fungi-*Sc* and *Sp*) were aligned and coloured according to the CLUSTALX colour scheme. Arrows above indicate the 11 residues that interact with FFAT in an NMR study [15], with the nature of interaction indicated below as: electrostatic (shown as + strong, ± weaker), nuclear Overhauser effects (13 overall in 6 different bond pairs involving 4 residues – each bond pair shown by the number of NOEs), and 9 hydrophobic interactions (shown as Ø), where two groups of four of these interactions are in hydrophobic pockets (shown as Ø––Ø—Ø—Ø). Numbers indicate the residues for human VAP-A and VAP-B. With the exception of the truncated human VAP-C, 10 of the interacting residues are all highly conserved from mammals to fungi to plants. M89 is the sole exception, varying to L in plants, fungi and Farinelli, one of two VAPs in flies.
(TIF)

Table S1 **All sequences with EFFDA-E or any of 127 combinations of allowed residues.** 4 sections depend on starting residues: A = DF…, B = DY…, C = EF…, D = EY…, each section containing all eukaryotic sequences containing any of the 32 variants of the FFAT-like motif starting with the two selected residues. Numbers in column 6 indicate the number of proteins with a specific motif. In column 7, C, O, RB, R3 and R11 refer to homologues of CERT, OSBP, rdgB, Rab3GAP1 and rabphilin11 respectively, and numbers in square brackets indicate where the list contains multiple homologues in the same family.
(PDF)

Table S2 **Defining FFAT-like motifs.** A. Criteria for FFAT-like motifs, and determining number of suboptimal elements. Top row: to be included in the list of possible motifs, sequences of 13 residues (7 in the body of the motif and 6 upstream) were found that contained at least 5 of the 6 features defined in the search pattern in the top row. Then, for all motifs, penalties were assigned to each element depending on how close they match the optimal. At each position, the strength of penalty for a particular substitution was judged primarily by the strength of its inhibitory effect on targeting, and secondarily by its rarity in alignments within families of proteins containing FFAT-like motifs. Typical penalties at most positions were 1 unit, except position 5 = 0.5 unit, acidic flank: 1.5 units. **Notes:**° Ø is any hydrophobic residue: CILMVW. * For the upstream flank, an estimation of its overall negative charge (Δ) was made that included the potential to develop such charge by phosphorylation: each residue in the flank was assigned a charge value: D/E = 1, S/T = 0.5, K/R = −1, all others = 0. Δ is the sum of these values, and can vary between +6 to −6. ‡ To allow scoring of unique variants and of mutations that we introduced, extreme substitutions were given extreme penalties, particularly in the flank, and positions 2 and 4. B. Overall numbers of human FFAT-like motifs identified with these criteria. This table examines the total pool of FFAT-like motifs in human proteins at PROSITE as having 5 of the 6 criteria in the top row of Table S2A (n = 3052). Among 15 with ≤1 suboptimal element, 12 had been identified before (ref [14]). Applying the cut-off of ≤2 suboptimal elements produces 62 new motifs (see Table S3 for detailed descriptions). An increase in cut-off (≥2.5) would lead to many more FFAT-like motifs being considered, as the number of motifs approximately doubles for every extra 0.5 sub-optimal element allowed.
(PDF)

Table S3 **Possible new FFAT-like motifs in 18 proteins.** New FFAT-like motifs (and flanking residues) from two groups of candidate proteins (1) human proteins containing motifs with 2 or less suboptimal elements (column 4, see Table S3), and (2) proteins of any species with motifs among the 127 simple variants of FFAT (see Table S1) were assessed by four criteria: (A) location in cytoplasm; (B) known not to form a helix in published crystallographic/NMR structures; (C) specific conservation across evolution of key FFAT residues compared to adjacent residues in orthologues of the protein. Poorly conserved residues in orthologues are underlined. Where residues in evolutionary distant orthologues fit FFAT better, they are in bold; (D) ≤2.0 sub-optimal elements, calculated as in Table S2. 21 motifs passed all four criteria; these appear in a section at the top bounded with red tram lines, and are marked with a tick in column 3. Three of these are already referred to in the text: AKAP110, AKAP220 and protrudin, leaving 18 new FFAT-like motifs. A second short section of the table shows the FFAT-like motifs from known VAP interactors GLTP, Orp3a, USP20 and RMD3 analyzed in the same manner. All other motifs failed one or more of the criteria, and are shown grouped according to the criterion they fail. Alternative names and information on the reasons for failing criteria are given for each protein.
(PDF)

Table S4 **Analysis of suboptimal elements in FFAT-like motifs described in this study.** Scoring criteria developed in Table S2 were applied to (A) the FFAT-like motifs identified in GLTPs and FAPPs in Figure 4, and (B) the FFAT-like motifs identified in Figure 3 in: StART proteins related to Edr2; ORPs in plants and other species; rabphilin11 homologues; and a variant of Opi1p.
(PDF)

Table S5 **FFAT-like motifs either expressed in this study or in known VAP-interactors.** This table provides detailed information on sequences listed in Table 1. † All sequences (°except protrudin, USP20 and RMD3, which aer included only for comparison) were cloned after GFP-myc. The accession numbers and species of origin (where not human) of the open reading frames are: Rab3GAP1 - 289547212; AKAP220 - 7671392; AKAP110 - 217416351; Orp1c: *Glycine max* - 164457637; Orp2a: *A. thaliana* - 7269100; Fapp2: *Monodelphis domestica­* - 312283618; Fapp2: *Hs* - 158706386; MAST205 - 112363080; Src - 4885609; Ypt11p - 82795261 (*S. cerevisiae*) and protrudin - 50557646. Sequences are coloured with acids red, S/T orange, basic residues blue. The core FFAT-like motifs are highlighted in light blue. Variations introduced for residues in this study are in bold and underlined. # is the number of amino acids from each target proteins expressed in this study. * “changes”: summarizes changes introduced to the natural motif. In dimers/tetramers changes were applied to all copies. ¶ “gen.±”: indicates the overall charge (D/E = −1; K/R = +1) in the flanks close to, but excluding, the FFAT-like motif. § “structure”: is the predicted (if known then **in bold**) structure in this region of the protein. “U” means unstructured or extended. Numbers refer to the residues involved in any main structural feature. ‡ S5 in this motif was substituted with A to prevent any inhibitory phosphorylation of that site. °FFAT-like motifs of protrudin, USP20 and RMD3 were not tested in this study, but are shown for comparison. An alternative FFAT-like motif in RMD3 is underlined.
(PDF)
