# Supplementary figures and images for: Analysis of the Key Elements of FFAT-Like Motifs Identifies New Proteins That Potentially Bind VAP on the ER, Including Two AKAPs and FAPP2
Source: PLoS One. 2012 Jan 19;7(1):e30455. doi: 10.1371/journal.pone.0030455 (PMC3261905; doi:10.1371/journal.pone.0030455)

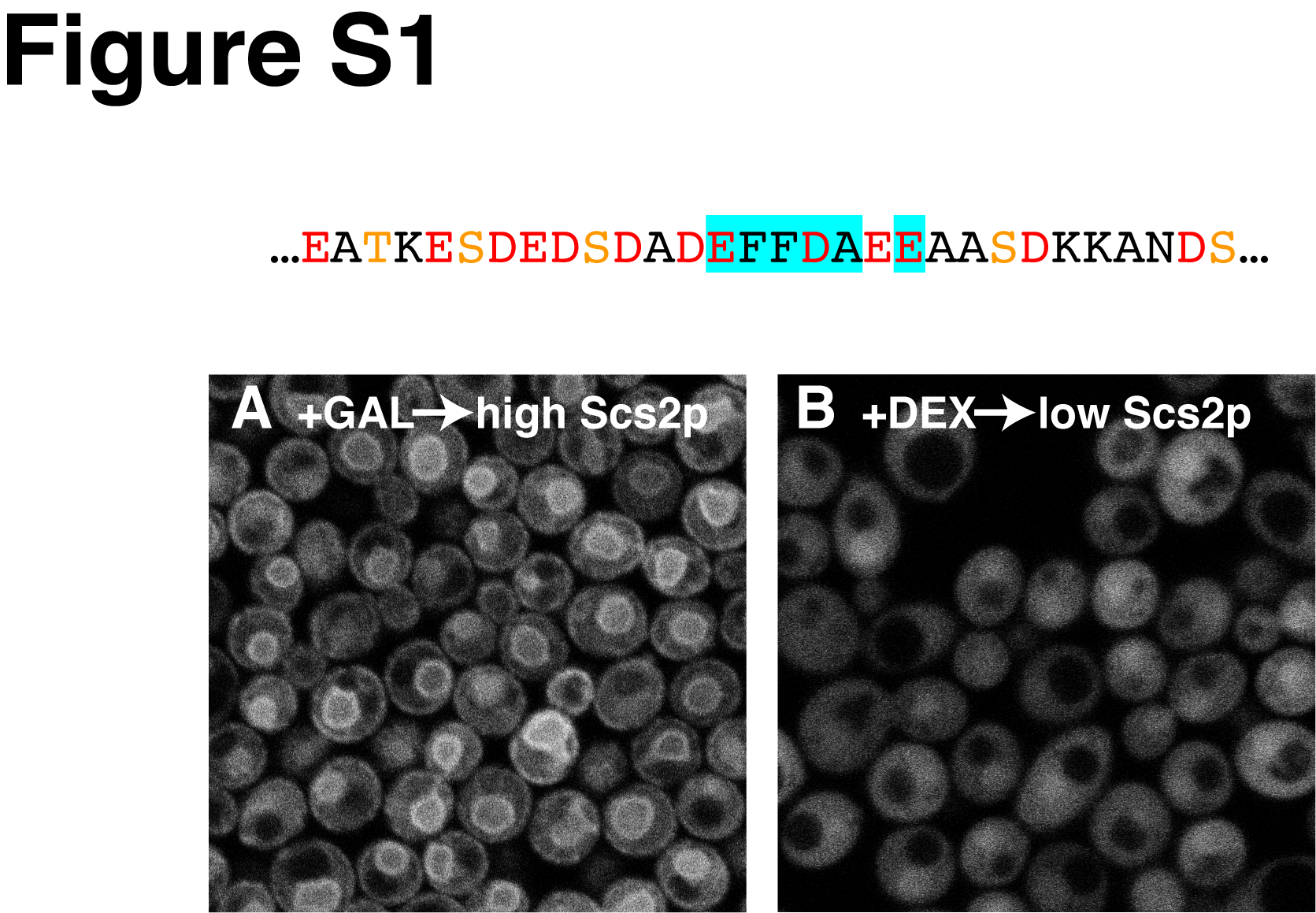

Supplement: Figure S1 — Relationship between FFAT motif targeting the ER and expression of Scs2p. The FFAT-containing region of Osh1p (residues 687–796) was expressed from pTL377 [14] in TLY251, which was grown either in galactose (+GAL) to induce maximal expression of yeast VAP (Scs2p), or in dextrose (+DEX) to repress expression. As in Figure 1, fluorescence was measured across nuclear profiles to produce targeting strength ratios, see Table 1. (TIF) [file pone.0030455.s001.tif]

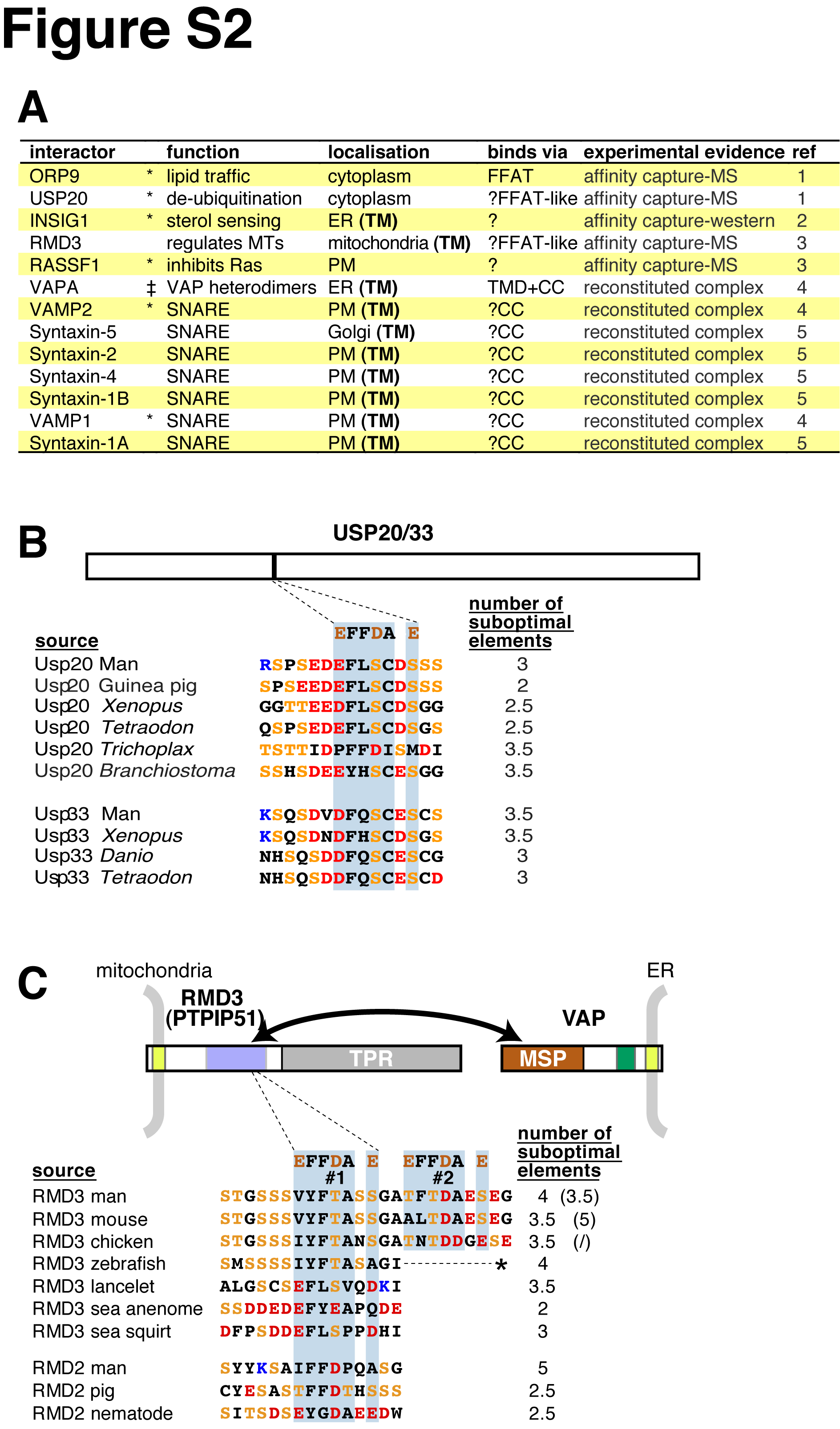

Supplement: Figure S2 — Possible additional FFAT-like motifs found by data mining. A. Interactors of VAPB currently listed at BIOGRID (http://thebiogrid.org/). The bottom 8 were found in targeted studies of VAP-SNARE interactions, and bind via the coiled coil and/or transmembrane domain. The top 5 were found in high-throughput studies. Of these, one has a known FFAT (ORP9), and two others have FFAT-like motifs that might mediate the interaction with VAP: USP20 and RMD3 (see parts B and C). References: 1. Sowa et.al. (2009) Cell 138: 389–403; 2. Gong et al. (2006) PNAS 103: 6154–6159; 3. Hutchins et al. (2010) Science 328: 593–599; 4. Nishimura et al. (1999) BBRC 254: 21–26; 5. Li et al. (2003) JBC 278: 19791–19797. B. A FFAT-like motif in USP20 is well conserved. An unstructured loop in human USP20 contains a FFAT-like motif that is marginally suboptimal (has more than 2 suboptimal elements in most but not all species), but is well conserved not only in all vertebrates but also in primitive metazoa such as Trichoplax. A version of the same motif appears in a more restricted group of USP33 homologues: vertebrates excluding birds. C. Molecular mechanism underlying the RMD3-VAP interaction. RMD3 (also called PTPIP51 or FAM82A2) on the outer mitochondrial membrane binds VAP on the ER across the ER-mitochondrial contact site [55]. The molecular basis for the interaction has not been studied, but the minimal VAP-binding sequence in human RMD3 (light blue) contains two adjacent FFAT-like motifs, both of which are sub-optimal, so would not appear in our list of most likely interactors (Table S3). The motif is well conserved in RMD3 orthologues, and is more optimal in sea squirt (Ciona intestinalis) and sea anemone (Nematostella vectensis). The latter motif meets all our criteria for a strong FFAT-like motif (see Table S3, second section). The motif is weakest in fish, which have a highly acidic tract in the downstream region (asterisk). In addition, RMD2 in some species (but not man) contains a FF [file pone.0030455.s002.tif]

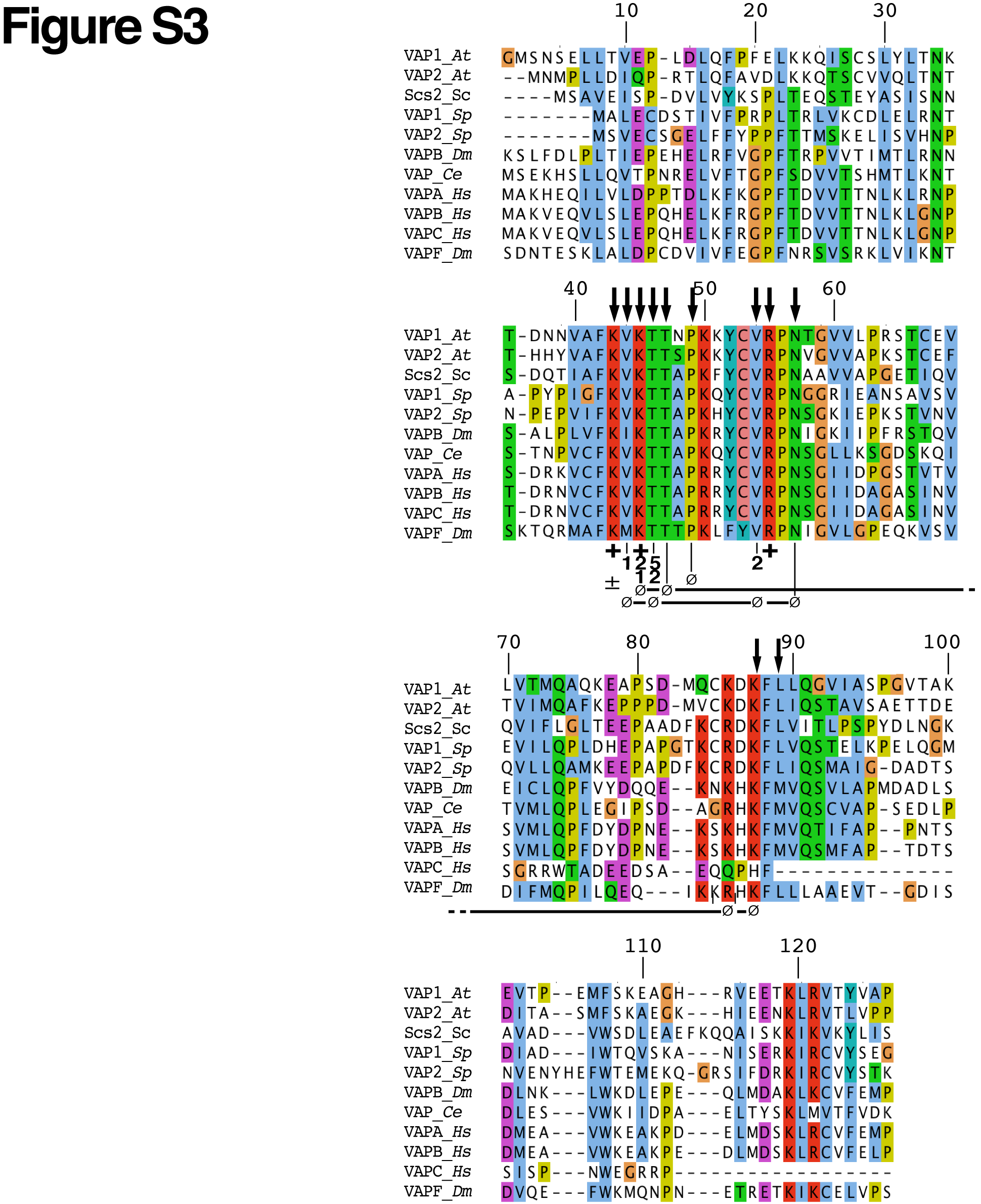

Supplement: Figure S3 — Residues in VAP that bind FFAT are conserved well throughout all eukaryotes. The amino-terminal MSP domains of all VAPs in the genomes of diverse model organisms (humans-Hs, flies-Dm, worms-Ce, plants-At, fungi-Sc and Sp) were aligned and coloured according to the CLUSTALX colour scheme. Arrows above indicate the 11 residues that interact with FFAT in an NMR study [15], with the nature of interaction indicated below as: electrostatic (shown as + strong, ± weaker), nuclear Overhauser effects (13 overall in 6 different bond pairs involving 4 residues – each bond pair shown by the number of NOEs), and 9 hydrophobic interactions (shown as Ø), where two groups of four of these interactions are in hydrophobic pockets (shown as Ø––Ø—Ø—Ø). Numbers indicate the residues for human VAP-A and VAP-B. With the exception of the truncated human VAP-C, 10 of the interacting residues are all highly conserved from mammals to fungi to plants. M89 is the sole exception, varying to L in plants, fungi and Farinelli, one of two VAPs in flies. (TIF) [file pone.0030455.s003.tif]
