# Supplementary material for: Analysis of the Key Elements of FFAT-Like Motifs Identifies New Proteins That Potentially Bind VAP on the ER, Including Two AKAPs and FAPP2
Source: PLoS One. 2012 Jan 19;7(1):e30455. doi: 10.1371/journal.pone.0030455 (PMC3261905; doi:10.1371/journal.pone.0030455)
Supplement: Table S1 — All sequences with EFFDA-E or any of 127 combinations of allowed residues. 4 sections depend on starting residues: A = DF…, B = DY…, C = EF…, D = EY…, each section containing all eukaryotic sequences containing any of the 32 variants of the FFAT-like motif starting with the two selected residues. Numbers in column 6 indicate the number of proteins with a specific motif. In column 7, C, O, RB, R3 and R11 refer to homologues of CERT, OSBP, rdgB, Rab3GAP1 and rabphilin11 respectively, and numbers in square brackets indicate where the list contains multiple homologues in the same family. (PDF) [file pone.0030455.s004.pdf]

Table S1. All sequences with EFFDA-E or any of 127 combinations of allowed residues.

TABLE S1A. Motifs starting DF…

1 & 2 E/D–F/Y	3 F or Y	4 D or E	5 A or C	7 D/E/S/T	count (6)	notes
DF	DFF	DFFD	DFFDA	DFFDA-D		
				DFFDA-E		
				DFFDA-S		
				DFFDA-T		
			DFFC	DFFDC-D		
				DFFDC-E	1	KSL10 (rice)
				DFFDC-S	1	CHCH-1 (mouse)
				DFFDC-T	1	Atg1-kinase (fungus)
		DFFE	DFFEA	DFFEA-D		
				DFFEA-E	1	Rhophilin-1 (mouse)
				DFFEA-S	1	Lim domain kinase-2 (bovine)
				DFFEA-T		
			DFFEC	DFFEC-D		
				DFFEC-E		
				DFFEC-S		
				DFFEC-T		
	DFY	DFYD	DFYDA	DFYDA-D		
				DFYDA-E		
				DFYDA-S		
				DFYDA-T		
			DFYDC	DFYDC-D		
				DFYDC-E		
				DFYDC-S		
				DFYDC-T	1	Hir3 (fungus)
		DFYE	DFYEA	DFYEA-D		
				DFYEA-E		
				DFYEA-S		
				DFYEA-T		
			DFYEC	DFYEC-D		
				DFYEC-E		
				DFYEC-S		
				DFYEC-T		

A. All eukaryotic sequences containing each of the 32 variants of the FFAT-like motif starting DF…

TABLE S1A. Motifs starting DY…

1 & 2 E/D-F/Y	3 F or Y	4 D or E	5 A or C	7 D/E/S/T	count (11)	notes
DY	DYF	DYFD	DYFDA	DYFDA-D		
				DYFDA-E	1	Slx1 endonuclease (fungal)
				DYFDA-S		
				DYFDA-T		
			DYFC	DYFDC-D	1	arylsuphatase (sea urchin)
				DYFDC-E		
				DYFDC-S	1	Mating pheromone 2 (Euplotes)
				DYFDC-T		
		DYFE	DYFEA	DYFEA-D		
				DYFEA-E	1	Ycf1 plastid protein
				DYFEA-S	1	Wit2 (Arabidopsis)
				DYFEA-T		
			DYFEC	DYFEC-D		
				DYFEC-E		
				DYFEC-S		
				DYFEC-T		
	DYY	DYYD	DYYDA	DYYDA-D		
				DYYDA-E		
				DYYDA-S	2	CQ072 (Hs unknown function)[2]
				DYYDA-T	2	Hemocyanin (octopus)[2]
			DYYDC	DYYDC-D		
				DYYDC-E		
				DYYDC-S		
				DYYDC-T		
		DYYE	DYYEA	DYYEA-D		
				DYYEA-E		
				DYYEA-S		
				DYYEA-T		
			DYYEC	DYYEC-D		
				DYYEC-E	1	Cation-Cl– cotransporter 1 (Arabidopsis)
				DYYEC-S	1	CHD-1 (Bombyx)
				DYYEC-T		

B. All eukaryotic sequences containing each of the 32 variants of the FFAT-like motif starting DY… (see footnote)

TABLE S1C. Motifs starting EF…

1 & 2 E/D–F/Y	3 F or Y	4 D or E	5 A or C	7 D/E/S/T	count (60)	notes
EF	EFF	EFFD	EFFDA	EFFDA-D	**2**	Vps13C (mouse) Dicer-like-2 (fungal)
				EFFDA-E	**29**	C[8] O[10] RB[7] R3[1] R11[2] Opi1
				EFFDA-S		
				EFFDA-T	**2**	O[2]
			EFFC	EFFDC-D	**2**	RB R3
				EFFDC-E	**1**	Mth-like 7 (fly)
				EFFDC-S		
				EFFDC-T		
		EFFE	EFFEA	EFFEA-D	**1**	Myosin IB light chain (Dicty.)
				EFFEA-E	**2**	Nop2[2]
				EFFEA-S		
				EFFEA-T		
			EFFEC	EFFEC-D		
				EFFEC-E		
				EFFEC-S	**5**	
				EFFEC-T		
	EFY	EFYD	EFYDA	EFYDA-D		
				EFYDA-E	**10**	O[5] Flipt1[2] R11[2] R07G3.7(worm)
				EFYDA-S	**4**	O[3] Rad2 DNA repair (yeast)
				EFYDA-T		
			EFYDC	EFYDC-D	**1**	nuclear phosphoprotein p8 (bovine)
				EFYDC-E		
				EFYDC-S		
				EFYDC-T		
		EFYE	EFYEA	EFYEA-D	**2**	alpha-1 acid glycoprotein (Hs lumenal)
				EFYEA-E	**1**	Formin-like 5 (rice)
				EFYEA-S		
				EFYEA-T	**1**	alpha-1 acid glycoprotein (lumenal)
			EFYEC	EFYEC-D	**3**	THEMIS1[3]
				EFYEC-E		
				EFYEC-S		
				EFYEC-T		

C. All eukaryotic sequences containing each of the 32 variants of the FFAT-like motif starting EF… (see footnote)

TABLE S1D. Motifs starting EY…

1 & 2 E/D-F/Y	3 F or Y	4 D or E	5 A or C	7 D/E/S/T	count (17)	notes
EY	EYF	EYFD	EYFDA	EYFDA-D	2	Vps13C (Hs) Mfh1 helicase (S. pombe)
				EYFDA-E	2	O[2]
				EYFDA-S		
				EYFDA-T		
			EYFC	EYFDC-D	1	Torso (tyrosine-kinase receptor, fly)
				EYFDC-E		
				EYFDC-S		
				EYFDC-T		
		EYFE	EYFEA	EYFEA-D		
				EYFEA-E	2	ALMT[2]
				EYFEA-S	5	Orc2[4] Mrt4 (fungal)
				EYFEA-T	1	CD46 (guinea pig)
			EYFEC	EYFEC-D		
				EYFEC-E		
				EYFEC-S		
				EYFEC-T		
	EYY	EYYD	EYYDA	EYYDA-D	2	Ferm/PDZ comtaining-1[2]
				EYYDA-E		
				EYYDA-S		
				EYYDA-T	1	beta-glucan synthesis-assoc pr.
			EYYDC	EYYDC-D		
				EYYDC-E		
				EYYDC-S		
				EYYDC-T		
		EYYE	EYYEA	EYYEA-D	1	COG-4 (worm)
				EYYEA-E		
				EYYEA-S		
				EYYEA-T		
			EYYEC	EYYEC-D		
				EYYEC-E		
				EYYEC-S		
				EYYEC-T		

D. All eukaryotic sequences containing each of the 32 variants of FFAT-like motif starting EY…

Footnote: Numbers in column 6 indicate the number of proteins with a specific motif. In column 7, C, O, RB, R3 and R11 refer to homologues of CERT, OSBP, rdgB, Rab3GAP1 and rabphilin11 respectively, and numbers in square brackets indicate where the list contains multiple homologues in the same family.
