# Supplementary material for: Analysis of the Key Elements of FFAT-Like Motifs Identifies New Proteins That Potentially Bind VAP on the ER, Including Two AKAPs and FAPP2
Source: PLoS One. 2012 Jan 19;7(1):e30455. doi: 10.1371/journal.pone.0030455 (PMC3261905; doi:10.1371/journal.pone.0030455)
Supplement: Table S2 — Defining FFAT-like motifs. A. Criteria for FFAT-like motifs, and determining number of suboptimal elements. Top row: to be included in the list of possible motifs, sequences of 13 residues (7 in the body of the motif and 6 upstream) were found that contained at least 5 of the 6 features defined in the search pattern in the top row. Then, for all motifs, penalties were assigned to each element depending on how close they match the optimal. At each position, the strength of penalty for a particular substitution was judged primarily by the strength of its inhibitory effect on targeting, and secondarily by its rarity in alignments within families of proteins containing FFAT-like motifs. Typical penalties at most positions were 1 unit, except position 5 = 0.5 unit, acidic flank: 1.5 units. Notes:° Ø is any hydrophobic residue: CILMVW. * For the upstream flank, an estimation of its overall negative charge (Δ) was made that included the potential to develop such charge by phosphorylation: each residue in the flank was assigned a charge value: D/E = 1, S/T = 0.5, K/R = −1, all others = 0. Δ is the sum of these values, and can vary between +6 to −6. ‡ To allow scoring of unique variants and of mutations that we introduced, extreme substitutions were given extreme penalties, particularly in the flank, and positions 2 and 4. B. Overall numbers of human FFAT-like motifs identified with these criteria. This table examines the total pool of FFAT-like motifs in human proteins at PROSITE as having 5 of the 6 criteria in the top row of Table S2A (n = 3052). Among 15 with ≤1 suboptimal element, 12 had been identified before (ref [14]). Applying the cut-off of ≤2 suboptimal elements produces 62 new motifs (see Table S3 for detailed descriptions). An increase in cut-off (≥2.5) would lead to many more FFAT-like motifs being considered, as the number of motifs approximately doubles for every extra 0.5 sub-optimal element allowed. (PDF) [file pone.0030455.s005.pdf]

Table S2. Defining FFAT-like motifs

A. Criteria for FFAT-like motifs, and determining number of suboptimal elements

	N-term charge Δ*	Main FFAT-like motif						
	-6 to 1=flank	1	2	3	4	5	6	7
Major features included in search patterns	≥ 1 D or E	any	FY	FYØ°H	DE ST	AC ST	any	DEST GNQY
optimal residue (no penalty)	Δ≥4	DE	F	FY	DE	AC	/	DE
Partial penalties	0.5 1	0.5		0.5			/	0.5
residue	Δ≥3 Δ≥-2	ST		Y				ST
**Typical penalty**	**1.5**	**1**	**1**	**1**	**1**	**0.5**	**/**	**1**
residue	Δ≥0.5	others	Y	Ø	ST	ST		GNQY
Moderate penalty	2				1.5	1		
residue	Δ≥-1				AIN	others		
Extreme penalty‡	2.5	1.5	2	1.5	2.5	2.5		1.5
residue‡	Δ<-1	K	others	others	others	DEKR	/	others

Top row: to be included in the list of possible motifs, sequences of 13 residues (7 in the body of the motif and 6 upstream) were found that contained at least 5 of the 6 features defined in the search pattern in the top row. Then, for all motifs, penalties were assigned to each element depending on how close they match the optimal. At each position, the strength of penalty for a particular substitution was judged primarily by the strength of its inhibitory effect on targeting, and secondarily by its rarity in alignments within families of proteins containing FFAT-like motifs. Typical penalties at most positions were 1 unit, except position 5 = 0.5 unit, acidic flank: 1.5 units.

° Ø is any hydrophobic residue: CILMVW.

* For the upstream flank, an estimation of its overall negative charge (Δ) was made that included the potential to develop such charge by phosphorylation: each residue in the flank was assigned a charge value: 1 for D/E, 0.5 for S/T, -1 for K/R. Δ is the sum of these values, and can vary between +6 to -6.

‡ To allow unique variants and mutations we introduced to be scored, extreme substitutions were given extreme penalties, particularly in the flank, and positions 2 and 4.

B. Overall numbers of human FFAT-like motifs identified with these criteria

Number of suboptimal elements	0	0.5	1	1.5	2	2.5	3	3.5	>3.5
Human FFAT motifs known before	4	2	6	2	0	0	0	0	0
Human FFAT-like motifs identified here	4	3	8	16	45	119	183	311	2683

This table examines the total pool of FFAT-like motifs in human proteins at PROSITE as having 5 of the 6 criteria in the top row of Table S2A (n=3052). Among 15 with ≤1 suboptimal element, 12 had been identified before (ref [14]). Applying the cut-off of ≤2 suboptimal elements produces 62 new motifs (see Table S3 for detailed descriptions). An increase in cut-off (≥2.5) would lead to many more FFAT-like motifs being considered, as the number of motifs approximately doubles for every extra 0.5 sub-optimal element allowed.
