# Supplementary material for: Analysis of the Key Elements of FFAT-Like Motifs Identifies New Proteins That Potentially Bind VAP on the ER, Including Two AKAPs and FAPP2
Source: PLoS One. 2012 Jan 19;7(1):e30455. doi: 10.1371/journal.pone.0030455 (PMC3261905; doi:10.1371/journal.pone.0030455)
Supplement: Table S3 — Possible new FFAT-like motifs in 18 proteins. New FFAT-like motifs (and flanking residues) from two groups of candidate proteins (1) human proteins containing motifs with 2 or less suboptimal elements (column 4, see Table S3), and (2) proteins of any species with motifs among the 127 simple variants of FFAT (see Table S1) were assessed by four criteria: (A) location in cytoplasm; (B) known not to form a helix in published crystallographic/NMR structures; (C) specific conservation across evolution of key FFAT residues compared to adjacent residues in orthologues of the protein. Poorly conserved residues in orthologues are underlined. Where residues in evolutionary distant orthologues fit FFAT better, they are in bold; (D) ≤2.0 sub-optimal elements, calculated as in Table S2. 21 motifs passed all four criteria; these appear in a section at the top bounded with red tram lines, and are marked with a tick in column 3. Three of these are already referred to in the text: AKAP110, AKAP220 and protrudin, leaving 18 new FFAT-like motifs. A second short section of the table shows the FFAT-like motifs from known VAP interactors GLTP, Orp3a, USP20 and RMD3 analyzed in the same manner. All other motifs failed one or more of the criteria, and are shown grouped according to the criterion they fail. Alternative names and information on the reasons for failing criteria are given for each protein. (PDF) [file pone.0030455.s006.pdf]

Table S3: Possible new FFAT-like motifs in 18 proteins.

New strong FFAT-like motifs meeting all criteria

Locus	FFAT like motif	pass or fail?	# sub-optimal elements	=Name of protein. + Comments on motif conservation/structure
AHNK2_HUMAN	lqeetiTFFDArEsf	✓	1.5	=Ahnak2. Reasonably conserved in most distant orthologues (VFFDAQE in birds). Predicted to be an unstructured loop.
AKA11_HUMAN	sdsevsEFFDSfDqf	✓	1	**=AKAP220**. #1: Precisely conserved in all orthologues. Predicted to be helical. #2: Precisely conserved in all orthologues. Weakly predicted to be helical.
AKA11_HUMAN	vssiedDFVTAfEhl		2	
ANR31_HUMAN	eessleTFVSAlEsl	✓	2	=Ankyrin rpt domain-containing pr-31. Well conserved in all orthologues (mammals only). Predicted to be helical.
ARHGC_HUMAN	lddsgeHFFDArEah	✓	1.5	=Rho GEF-12. Well conserved to most distant orthologue (frog: QFFDAQE).Predicted to be in a long unstructured loop.
CD19_HUMAN	dseegsEFYENdSnl	✓	2	=B lymphocyte antigen CD19. Conserved in all mammals. Predicted in a long unstructured loop.
CQ053_HUMAN	eefedeDFLSAvEda	✓	1.5	=Uncharacterized protein C17orf53. Well conserved in all orthologues. Predicted to be helical.
DVL3_HUMAN	selettSFFDSdEdd	✓	1.5	=Dishevelled 3. Well conserved in vertebrates. Not conserved in flies: SLFGTES. Predicted to be in an unstructured loop.
FA73B_HUMAN	sltsedSFFSAtElf	✓	1.5	=C9orf54. Well conserved in mammals. SFVSTAE in birds. F3→A in insects. Predicted to he helical.
FLIP1_HUMAN	tsptseEFFSStTvi	✓	2	=Filamin-A-interacting pr-1. Well conserved in all orthologues, DFFSSAT in frog. Predicted to be in a long unstructured loop.
FRPD1_HUMAN	dgssdeEYYDAaDkl	✓	1.5	=FERM and PDZ domain-containing pr-1 (FRMPD1). Well conserved. EFFDARD in fish (most distant orthologues). Predicted to be in loop.
FRPD1_MOUSE	ggssdeEYYDAaDkl		2	
NADAP_HUMAN	knwedeDFYDSdDdt	✓	2	=Kanadaptin. Conserved in most eukaryotes *e.g.* DYYDSDE in *Trichoplax*, but not in flies & plants: DFYDRTK. Predicted to be in a short loop.
RASF3_HUMAN	eedaedFFFTArTsf	✓	2	=Ras association domain-containing pr-3 (RASSF3). Reasonably conserved, *e.g.* LFYTART in frog. Weakly predicted to form a helix.
RHPN1_HUMAN	eldredDFCEAaEap	✓	1.5	=Rhophilin-1. Well conserved, *e.g.* fish DFCEVAE sea squirt: DFFDIVE + flank more acidic. Lies after helix 12 of BRO1 domain.* Absent in Rhpn-2.
RHPN1_MOUSE	qleredDFFEAtEap		1	
S22AF_HUMAN	eseeeeEFYDAdEet	✓	0.5	=Solute carrier (SLC)-22 member 15/Flipt**.** Conserved to most distant orthologue (lizard: EFFDADE). Weakly predicted to be helical.
S22AF_MOUSE				
SCN4A_HUMAN	geddfeMFYETwEkf	✓	2	=Sodium channel protein type 4/10 subunit alpha. Well conserved in mammals and some fish, but in other fish T5→Ø. Not conserved in flies (MYYEIWQ). Predicted to be helical.
SCNAA_HUMAN	seddfdMFYETwEkf		2	

SPKAP_HUMAN	lssieeDFLTAsEhl	✓	2	**=AKAP110.** Perfectly conserved in all orthologues (incl. fish). Weakly predicted to be helical. Well conserved in all orthologues incl. fish. Predicted to be helical.
SSH2_HUMAN	fstdriDFFSAlEkf	✓	2	=Slingshot homolog 2. Well conserved to birds/fish. F3L in frog. Motif not in fly SSH. Predicted helical.
TACC1_HUMAN	epeedlEYFECsNvp	✓	2	=Transforming acidic coiled-coil-containing pr-1. Motif stronger in frogs E**F**FECTS, but missing in fish orthologues. Predicted to be in a long loop.
THMS1_BOVIN	plsqegEFYECeDeh	✓	2	=Thymocyte-expressed molecule involved in selection (THEMIS). Well conserved to most distant orthologue: EFYECAD in fish. E1K in birds. Predicted to be in an unstructured loop.
THMS1_HUMAN	plsqegEFYECeDer		2	
THMS1_MOUSE	plsqegEFYECeDeh		2	
VPS13A_HUMAN	eddseeEFFDApCsp	✓	1.5	=Vps13A / chorein. Conserved in all Vps13A orthologues, *e.g.* EFYDAPT in frog. Predicted to be in long extended loop. In same position as motif in Vps13C (see above).
VP13C_HUMAN	esesddEYFDAeDge		1	=Vps13C. Well conserved even to fungi: EFVDAVS in *S. pombe*. Predicted to be in long extended loop. In same position as motif in Vps13A (see above).
VP13C_MOUSE	esesdeEFFDAeDgd		0	
ZFY27_HUMAN	eaepdeEFKDAiEet	✓	1.5	**=Protrudin.** (also called SPG33). Conserved in all orthologues incl. fish. Predicted helical.

Known VAP interactors

GLTP (*H. sapiens*)	avshlpPFFDClGsp	(B & D)	3.5	See text. Conserved in fish, not invertebrates (see Fig 3A). **BUT:** PFFD in 3/10-helix (helix 2) in crystal structure (1SWX).
Orp3a (*A. thaliana*)	gqkfapKWFDEtEev	B,C &D	6	See text. Not well conserved, *e.g.* RWFNLTD in maize.
USP20 (guinea pig)	SpseedEFLSCdSss	✓	2	Reasonably conserved in all vertebrates. Weaker motif in closest homologue USP33.
RMD3 (*Nematostella. vectensis*)	ssddedEFYEApQde	✓	2	=PTPIP51. Reasonably well conserved, *e.g.* VYFTASS in human (see Figure S3). In unstructured region. Also weaker motif in RMD2

Fail criterion (A): Not located in cytoplasm

A1AG_RABIT	speqleEFYEAlTcl	A	2	= alpha-1 acid glycoprotein. Extracellular.
A1AG1_HUMAN	tkeqlgEFYEAlDcl	A	2	
A1AG2_HUMAN	tkeqlgEFYEAlDcl	A	2	
ACCN2_HUMAN	tmdsdlDFFDSySit	A	1.5	=Amiloride-sensitive cation channel 2. Extracellular.
ALMT1_ARATH	lqdfgdEYFEArEkg	A	2	=Aluminium-activated malate transporter 1/2 (*Arabidopsis*). Extracellular.
ALMT2_ARATH	lqefgdEYFEAtEdg			
FA69C_HUMAN	tgdedcNFFDCfSrc	A	2	=C18orf51. Extracellular.
PCDB9_HUMAN<br>PCDBA_HUMAN	naevsySFFDAsEdi	A	2	=Protocadherin beta-9/10. Extracellular.
YCF1_OENEH	lkkelkDYFEAqElf	A & D	2.5	=Ycf1 (plastid). (and poorly conserved: *e.g.* DYFGAQG in closest orthologue)

Fail criterion (B): occur in helix of known crystal/NMR structure

AP3M1_HUMAN	sqsvcdYFFEAqEka	B	2	=μ-adaptin 3A. Not as well conserved as surrounding residues, *e.g.* YYLEAQR in *Tribolium*. In helix 1 of MU-1 (2VGL)
KAT1_HUMAN	eviiiePFFDCyEpm	B	2	=Kynurenine aminotransferase I. Fully conserved in vertebrates. However, forms part of internal substrate pocket.
LCK_HUMAN	lrsvleDFFTAtEgq	B	2	=Lck tyrosine kinase. Absolutely conserved in ALL vertebrate Src-like kinases (±F3Y), invertebrates lose 2nd half: *e.g.* EFFVMEG in sea urchin. Structure: 1st half is in helix 13 of kinase domain.
MAOX_HUMAN	yddfldEFMEAvSsk	B	2	=NADP-dependent malic enzyme. In helix 16 of structure. Also: residue 4 not conserved in fish: EFMKAIT.
NOM1_HUMAN	timtseDFLDAfEkl	B	2	=Nucleolar MIF4G domain-containing protein 1. In helix 2 of MA3 domain.
NOP2_SCHPO	svseavEFFEAnEmp	B	1	=ribosomal RNA methyltransferase (nucleolar protein 2). Well conserved, EFLEANE in human. But, lies in helix 3 of methyltransferase domain.
NOP2_YEAST	spaeamEFFEAnEia	B	1.5	
S14L6_HUMAN	giellqEFFSAlEan	B	2	=Putative SEC14-like protein 6. In helix 7 of conserved Sec14 structure.
TBAL3_HUMAN	egmeeaEFLEArEdl	B	1.5	=Tubulin alpha chain-like 3. In helix 16 of tubulin alpha-1C domain
ZBT11_HUMAN	ctqcekSFFEArDlr	B	2	=Zinc finger and BTB domain-containing pr-11. Well conserved in orthologues: *e.g.* RFYEAKD in fish. But 2nd half in helix of 7th C2H2 Zn finger.

Fail criterion (C): core residues (FFD) not well conserved in orthologues

ATG1_ASPOR	ermnfsDFFDCdTit	C	2	=Autophagy-related pr-1 (*Aspergillus oryzae*). Poor conservation *e.g.* DFFQNGV in other *Aspergillus*
ATMIN_HUMAN	idfdieEFFSAsNiq	C	2	=ATM/ATR-substrate CHK2-interacting Zn finger pr. Conserved in mammals/birds, but not in distant orthologues, *e.g.* EFLNATH in fish.
CDR1_HUMAN	dfledpDFLEAiDlr	C	1.5	=Cerebellar degeneration-related antigen 1. Along with 5 DFLED repeats. Not conserved in close orthologues, *e.g.* DFLEAMN in horse.
CEBPZ_HUMAN	esddeeNFIDAnDde	C	2	=CCAAT/enhancer-binding pr. zeta. Only conserved in mammals. CFKDQEE in birds.
CHCH1_MOUSE	crkeiqDFFDCsSra	C	2	=Coiled-coil-helix-coiled-coil-helix domain-containing pr-1. Very poorly conserved, *e.g.* GFLDCAA in chimp
DCL2_PHANO	ivsdveEFFDAdDgg	C	1	=Dicer-like protein 2 (*Phaeospheria*). Only conserved in closest orthologues in *leptospheria* and *pyrenophora*.
DSEL_HUMAN	teleidSFVDAcEwk	C	2	=Dermatan-sulfate epimerase-like pr. Not well conserved, *e.g.* SLLDACE in fish.
E41LA_HUMAN	dcnetsFFFEArSkt	C	2	=Band 4.1-like protein 4A. Not well conserved as surrounding residues, *e.g.* FFFKAPN in fish.

ERO1A_HUMAN	hddssdNFCEAdDiq	C	2	=Endoplasmic oxidoreductin-1-like pr. Key residues not conserved. QFCDIDD in sea squirt.
FA21A_HUMAN	dgddddDFFSApHsk	C	2	=WASH complex subunit FAM21A/B/C. Not conserved in close orthologues; NFFMPSS in rat
FA21B_HUMAN	dgddddDFFSApHsk	C	2	
FA21C_HUMAN	dgddddDFFSApHsk	C	2	
FH5_ORYSJ	svdtgeEFYEAeEdw	C	1	=Formin-like protein 5 (rice). Poorly conserved, *e.g.* EIFSEAE in *Arabidospsis lyrata.*
FKBP6_HUMAN	feiellDFLDCaEsd	C	2	=FK506 binding pr-6. Conserved in mammals, less so in fish: DFLDSGQ. Predicted to be at end of beta strand 7 and in loop following.
GSTA4_HUMAN	gvefdeEFLETkEql	C	2	=Glutathione S-transferase A4-4. Key residue not conserved compared to nearby residues. EMIETRE in frog.
HELB_HUMAN	eedeesVFIDAeElc	C	2	=Helicase B. Better in some mammals (**E**FVDAEE). But less conserved in fish: EFLDMKE. Motif absent in birds. Weakly predicted to be helical.
HOT_HUMAN	veptdsSFMEAiEfa	C	2	=Hydroxyacid-oxoacid transhydrogenase. Poor conservation compared to surrounding residues. SFKAAID in fish.
IL1B_HUMAN	ifeeepIFFDTwDne	C	2	=Interleukin-1 beta (precursor). Poorly conserved in mammals, *e.g.* dog has only acidic tract and no other discernible elements.
JMJD4_HUMAN	ssdwlnEFWDAlDvd	C	2	=JmjC domain-containing pr-4. Only 1st half of motif conserved in invertebrates: *e.g.* EFWDHRQ in sea urchin.
KSL10_ORYSJ	litvydDFFDCpEis	C	1.5	=Ent-kaurene synthase-like 10 (rice), Poorly conserved, *e.g.* DFFDGEG in closest homologue also in rice.
LIMK2_BOVIN	afskleDFFEAlSly	C	2	=LIM domain kinase 2. Poorly conserved, *e.g.* DSFEALS in closest orthologue (horse)
MLCB_DICDI	teaeitEFFEAaDpn	C	0.5	=Myosin-IB light chain (slime mold) Poorly conserved, *e.g.* EFLAAAD in close orthologue (*D. fasciculatum*)
MTH7_DROME	maiirdEFFDCdEmi	C	1.5	=Methusaleh-7 (fruit fly). Poorly conserved, *e.g.* PFLSCDE in close orthologue (*D. yakuba*)
ORC2_HUMAN	tsdlveEYFEAhSss	C	2	=Origin recognition complex subunit 2. E4 poorly conserved beyond mammals. *e.g.* Q4 in fish. Weakly predicted to be helical.
NRBF2_HUMAN	eldvdaDFVETsElw	C	2	=Nuclear receptor-binding factor 2. Key residue not conserved: T5→K *e.g.* in mouse.
PK1IP_HUMAN	gdeeviRFFDCdSlv	C	2	=p21-activated protein kinase-interacting protein 1 (hPIP1). Key residue not conserved: F2→I *e.g.* in mouse
PKHM3_HUMAN	alevteEFFSTlDsn	C	2	=Pleckstrin homology domain-containing family M member 3. Conserved in mammals, but S4→N in birds. Predicted to be helical.
R51A1_HUMAN	dseddsDFCESeDnd	C	1.5	=RAD51-associated pr-1. Poorly conserved in mammals, *e.g.* rat has DFDESEE.
RTL1_HUMAN	dsdhseTFYECpSta	C	1.5	=Retrotransposon-like pr-1 (PEG11) .Poorly conserved, *e.g.* GFYGYGY in pig

TSYL2_HUMAN	dfmettDYFETtDne	C	2	=Testis-specific Y-encoded-like pr-2. Key residue not conserved: Y2→C *e.g.* in dog.
YG044_BOVIN	pvgseeEFYDCpDyy	C	1.5	=Uncharacterized protein ENSP00000370281 homologue. Poorly conserved, *e.g.* F2 substituted in closest orthologues e.g. F2→L in pig.
YRO7_CAEEL	dsepdqEFYDAqEqe	C	1	=Uncharacterized protein R07G3.7 (*C. elegans*). Poorly conserved, *e.g.* AFLEAKK in close orthologue in *C. briggsae*

Fail criterion (D): more than 2 suboptimal elements in motif

COG4_CAEEL	mtmqdvEYYEAhDpf	D &C	3	=Conserved Oligomeric Golgi component 4 (*C. elegans*). Conserved in genus, but not in related *Brugia*: ADFEAED
CQ072_HUMAN	dvppasDYYDAeSll	D	3.5	=C17orf72. Conserved in mammals but not fish orthologues, *e.g.* EHYEVER.
CQ072_MOUSE	dvppasDYYDAeSlp			
HCYA_ENTDO	rlladsDYYDAwTdn	D &C	3.5	=Hemocyanin A-type (Octopus). Not well conserved, *e.g.* NYYYVWR in *Aplysia*.
HCYG_ENTDO	rlladsDYYDAwTdn			
MCP_CAVPO	sdvqvfEYFEAvTys	D &C	3	=Membrane cofactor precursor (*Cavia porcellus*). Not well conserved, *e.g.* QYREAVI in Chinese hamster.
MFH1_SCHPO	ngysvdEYFDAnDsn	D &C	2.5	=ATP-dependent DNA helicase (*S. pombe*). Very poorly conserved, EFMNRNV in *S. japonicus*.
MRT4_CANGA	tlDtvkEYFEAySrl	D &C	3	=mRNA turnover protein 4 (*Candida*). Poorly conserved, EYFKSYV in *S. cerevisiae*.
ORC2_MOUSE	asdlveEYFEAhSss	D	2.5	=Origin recognition complex subunit 2. E4 poorly conserved beyond mammals. Q4 found in fish. Weakly predicted to be helical
ORC2_RAT	agdlveEYFEAhSss		2.5	
ORC2_XENLA	asnlvEEYFEAhSss		3	
RAD2_YEAST	heknyvEFYDAeSil	D &C	2.5	=DNA repair protein RAD2 (*S. cerevisiae*). Poorly conserved compared to adjacent residues, *e.g.* ECYMQDD in *C. tropicalis*.
SLX1_BOTFB	keqelvDYFDAdEfh	D &C	2.5	=structure-specific endonuclease subunit slx1 (noble rot fungus). Very poorly conserved – only in one species.
WIT2_ARATH	eeiireDYFEAlSsr	D &C	2.5	=WPP dom.-interacting tail-anchored pr-2 (*Arabidopsis*). Poorly conserved, *e.g.* EHGEAVS in *A. lyrata*
YEOB_SCHPO	aattdyEYYDAdTpt	D &C	3	=uncharacterized beta-glucosidase (*S. pombe*). Poorly conserved, EWYDPDA in *S. japonicus*.

New FFAT-like motifs (and flanking residues) from two groups of candidate proteins (1) human proteins containing motifs with 2 or less suboptimal elements (column 4, see Table S3), and (2) proteins of any species with motifs among the 127 simple variants of FFAT (see Table S1) were assessed by four criteria:
(A) location in cytoplasm;
(B) known not to form a helix in published crystallographic/NMR structures;
(C) specific conservation across evolution of key FFAT residues compared to adjacent residues in orthologues of the protein. Poorly conserved residues in orthologues are underlined. Where residues in evolutionary distant orthologues fit FFAT better, they are in bold;
(D) ≤2.0 sub-optimal elements, calculated as in Table S2.

21 motifs passed all four criteria; these appear in a section at the top bounded with red tram lines, and are marked with a tick in column 3. Three of these are already referred to in the text: AKAP110, AKAP220 and protrudin, leaving 18 new FFAT-like motifs.

A second short section of the table shows the FFAT-like motifs from known VAP interactors GLTP, Orp3a, USP20 and RMD3 analyzed in the same manner. All other motifs failed one or more of the criteria, and are shown grouped according to the criterion they fail. Alternative names and information on the reasons for failing criteria are given for each protein.
