# Supplementary material for: Analysis of the Key Elements of FFAT-Like Motifs Identifies New Proteins That Potentially Bind VAP on the ER, Including Two AKAPs and FAPP2
Source: PLoS One. 2012 Jan 19;7(1):e30455. doi: 10.1371/journal.pone.0030455 (PMC3261905; doi:10.1371/journal.pone.0030455)
Supplement: Table S4 — Analysis of suboptimal elements in FFAT-like motifs described in this study. Scoring criteria developed in Table S2 were applied to (A) the FFAT-like motifs identified in GLTPs and FAPPs in Figure 4, and (B) the FFAT-like motifs identified in Figure 3 in: StART proteins related to Edr2; ORPs in plants and other species; rabphilin11 homologues; and a variant of Opi1p. (PDF) [file pone.0030455.s007.pdf]

TABLE S4. Analysis of suboptimal elements in FFAT-like motifs described in this study

A. GLTP/FAPP2

				# suboptimal elements
#1	GLTP	fish: sablefish	MTLLLDNQ	/
	GLTP	fish: lancelet	mAFFTDYEHQ	5
	GLTP	fungus: Debaryomyces	msTFFDEMKKS	5.5
	GLTP	sea squirt: Oikopleura	maadntCYFDQMKRS	6
	GLTP	fungus: stem rust	maadntCYFDQMKRS	6
	GLTP	protist: Capsaspora	apqqpaTFFDTmPys	3.5
	FAPP2	sponge: Amphimedon	atidydTFFSKlPhk	5
	FAPP2	sea squirt: Ciona	nedeplTFFSAmLhs	2.5
	FAPP2	lizard: anole	tnkdfkTFFSAmSir	3
	FAPP2	mammal: human	gkevipTFFSTmNts	4
	FAPP2	mammal: opossum	eeeevqTFFSAmNts	2
	FAPP2	bird: zebra finch	nendspTFFSVmSnr	3.5
	FAPP2	sea squirt: Oikopleura	seivekMFFNEiEhs	5.5
#2	GLTP	mammal: human	kqietgPFLEAvShl	4
	GLTP	fish: sablefish	kaidtkLFLESvShi	4.5
	GLTP	fish: lancelet	gkvetgPFLLAsLrl	5.5
	GLTP	fungus: Debaryomyces	kkidtaSFLEAsEsl	3
	GLTP	sea squirt: Oikopleura	egihveQFLSAsRsy	5.5
	GLTP	fungus: stem rust	egvdtlAFLEAcEdl	3
	GLTP	protist: Capsaspora	ygiatlPFLNAvHat	6
	FAPP2	sponge: Amphimedon	egiptdSFLQCcSdl	5
	FAPP2	sea squirt: Ciona	ggidsiSFLLScEgi	4.5
	FAPP2	lizard: anole	egipteEFLRScYei	5.5
	FAPP2	mammal: human	sgipteAFLAScCav	5.5
	FAPP2	mammal: opossum	sgipteAFLEScYav	4.5
	FAPP2	bird: zebra finch	egipteEFLEScYai	3.5
	FAPP2	sea squirt: Oikopleura	lelpvnQFLSAaAdl	5
#3	GLTP	mammal: human	avshlpPFFDClGsp	3.5
	GLTP	fish: sablefish	svshipSFFDClGss	3
	GLTP	fish: lancelet	aslrllPFFDMlGpt	4.5
	GLTP	fungus: Debaryomyces	asesliKLFDLlGss	5.5
	GLTP	sea squirt: Oikopleura	asrsylEFYDLfGgt	4
	GLTP	fungus: stem rust	acedlvRLFDLfGsk	5
	GLTP	protist: Capsaspora	avhatiPLFDTlGml	5
	FAPP2	sponge: Amphimedon	ccsdllPFFDAlSpt	3
	FAPP2	sea squirt: Ciona	scegiiPFLDTiGst	5
	FAPP2	lizard: anole	scyeivPVLDKlGpt	7.5
	FAPP2	mammal: human	sccavvPVLDKlGpt	7.5
	FAPP2	mammal: opossum	scyavvPVLDKlGpt	7.5
	FAPP2	bird: zebra finch	scyaivPVLDKlGpt	7.5
	FAPP2	sea squirt: Oikopleura	aaadllLIIDKlGsk	7.5

B. Other FFAT-like motifs in ORPs, rabphilin-11 homologues and an Opi1p variant

			# suboptimal elements
	At2g28320	slvdldEFFDVpEps	2
	At3g54800	lndasdEFFDVpEpn	2
	At4g19040=Edr2	dsddddEFQIAeSeq	2.5
	At5g45560	dsddddEFQIPdSep	3.5
	Orp1c At	fdeeenTFFDTrDfl	1
	Orp1c Gm	tddddnAFFDTrDil	1.5
	Orp1c Os	tdddenIYFDTrDfl	2.5
	Orp1c Zm	tddeanLYFDTrDfl	3
	Orp1c1 Vv	tddddnTFFDTrDfl	1
	Orp1c2 Vv	ddddddEFFDTqDfl	0.5
	Orp1c Pi	daddenLFVDAqDyl	2
	Orp1d At	sdeddvPYFDTnDil	3
	Orp1d Os	tdedevMYFDTrDfl	2.5
	Orp1a At	sdddneQFDEAeEem	2.5
	Orp2a1 Os	sdedefHFYDTrQsf	2.5
	Orp2a2 Os	tcedesTFFDAaDyf	0.5
	Orp2a Vv	sdeeetSFFDTrEdf	1
	Orp2a At	seedepSFHDTkEff	2.5
	Orp3a At	gqkfapKWFDEtEev	6
ORPs from other species	*A. gossypii*	asifseVFFDAlDdn	2
	C. intestinalis	flsdedEFYDAiSgd	1.5
	T. gondii	eddddvEFFECeDqe	0
	B. bovis	cghlddEFFECeDia	1
	T. guttata	silseeQFYDAvSds	2.5
	C. parvum	sesteeIFYDAfSdf	2
	C. muris	tseetdtFYDAlSdi	1.5
	T. parva	eskldtkFYEtaSte	4
Rab11bp	*H. sapiens*	sesdteEFYDApEdv	0.5
	T. nigroviridis	dtsdteEFYDApEdn	0.5
	At5g53500	sqeedlQFFDAnEem	1.5
	At5g24320	eeeeesRFFDAhEei	1
	At1g64610	deededRFFDApEvv	1
	At1g48870	evvvddLFFDSsDvl	2
	At5g42010	neeeedWFSDArEev	2
	At5g02430	fedddddHFFDSsNri	2.5
	At2g37670	ededddRFFEThDrl	1.5
Opi1	*A. gossypii*	eeedgeQYFDAsEti	2

Scoring criteria developed in Table S2 were applied to (A) the FFAT-like motifs identified in GLTPs and FAPPs in Figure 4, and (B) the FFAT-like motifs identified in Figure 3 in: StART proteins related to Edr2; ORPs in plants and other species; rabphilin11 homologues; and a variant of Opi1p.
