# Supplementary material for: Analysis of the Key Elements of FFAT-Like Motifs Identifies New Proteins That Potentially Bind VAP on the ER, Including Two AKAPs and FAPP2
Source: PLoS One. 2012 Jan 19;7(1):e30455. doi: 10.1371/journal.pone.0030455 (PMC3261905; doi:10.1371/journal.pone.0030455)
Supplement: Table S5 — FFAT-like motifs either expressed in this study or in known VAP-interactors. This table provides detailed information on sequences listed in Table 1. † All sequences (°except protrudin, USP20 and RMD3, which aer included only for comparison) were cloned after GFP-myc. The accession numbers and species of origin (where not human) of the open reading frames are: Rab3GAP1 - 289547212; AKAP220 - 7671392; AKAP110 - 217416351; Orp1c: Glycine max - 164457637; Orp2a: A. thaliana - 7269100; Fapp2: Monodelphis domestica­ - 312283618; Fapp2: Hs - 158706386; MAST205 - 112363080; Src - 4885609; Ypt11p - 82795261 (S. cerevisiae) and protrudin - 50557646. Sequences are coloured with acids red, S/T orange, basic residues blue. The core FFAT-like motifs are highlighted in light blue. Variations introduced for residues in this study are in bold and underlined. # is the number of amino acids from each target proteins expressed in this study. * “changes”: summarizes changes introduced to the natural motif. In dimers/tetramers changes were applied to all copies. ¶ “gen.±”: indicates the overall charge (D/E = −1; K/R = +1) in the flanks close to, but excluding, the FFAT-like motif. § “structure”: is the predicted (if known then in bold) structure in this region of the protein. “U” means unstructured or extended. Numbers refer to the residues involved in any main structural feature. ‡ S5 in this motif was substituted with A to prevent any inhibitory phosphorylation of that site. °FFAT-like motifs of protrudin, USP20 and RMD3 were not tested in this study, but are shown for comparison. An alternative FFAT-like motif in RMD3 is underlined. (PDF) [file pone.0030455.s008.pdf]

Table S5. FFAT-like motifs either expressed in this study or in known VAP-interactors

Source	#	position	Sequence†	changes*	gen.±¶	structure§
Osh1 110 aa	110	687-796	ESTNTLEEIVKFIEATKESDEDSDADEFFDAEEAASDKKANDSED	none	-18+4	U:666-793
Px domain Bem1p	148	266-413	(negative control)	none		(neg control)
Rab3GAP1-wt	26	570-595	EVGKSWDSWSDSEEEFFECLSDTEEL	none	-8+2	U:537-647
Rab3GAP1-4A	26	570-595	EVGKSWDSWSDSEEEFFACLSDTEEL	E4A	-8+2	U:537-647
Rab3GAP1-4Ae	26	570-595	EVGKEWDEWEDEEEEFFACLSDTEEL	E4A + 4E	-12+2	U:537-647
AKAP220-wt	29	343-371	DDIEDSDSEVSEFFDSFDQFDELEQTLET	none	-10+0	H:349-373
AKAP220–5A	29	343-371	DDIEDSDSEVSEFFDAFDQFDELEQTLET	S5A	-10+0	H:349-373
AKAP220–5E	29	343-371	DDIEDSDSEVSEFFDEFDQFDELEQTLET	S5E	-10+0	H:349-373
AKAP110-4D	32	199-230	LEDDTNCSLSSIEEDFLTASEHLEEESEVDES	none	-11+0	H:208-224
AKAP110-4D	32	199-230	LEDDTNCSLSSIEEDFLDASEHLEEESEVDES	T4D	-11+0	H:208-224
AKAP110-4A	32	199-230	LEDDTNCSLSSIEEDFLAASEHLEEESEVDES	T4A	-11+0	H:208-224
Orp1c:Gm-wt	34	315-348	SEDDNERNDAAEEETDDDDNAFFDTRDILSSSSF	none	-12+2	U:295:406
Orp1c:Gm-AA	34	315-348	SEDDNERNDAAEEETDDDDNAAADTRDILSSSSF	F2A F3A	-12+2	U:295:406
Orp1c:Gm-1M	34	315-348	SEDDNERNDAAEEETDDDDNMFFDTRDILSSSSF	A1M	-12+2	U:295:406
Orp1c:Gm-1H	34	315-348	SEDDNERNDAAEEETDDDDNHFFDTRDILSSSSF	A1H	-12+2	U:295:406
Orp1c:Gm-1K	34	315-348	SEDDNERNDAAEEETDDDDNKFFDTRDILSSSSF	A1K	-12+2	U:295:406
Orp2a:At-wt	32	297-328	DGKQEFEDVSEEDEPSFHDTKEFFNEPNIGSE	none	-10+2	U:254-355
Orp2a:At-x2	32	2x 297-328	DGKQEFEDVSEEDEPSFHDTKEFFNEPNIGSE	none	-10+2	U:254-355
Orp2a:At-x2-AA	32	2x 297-328	DGKQEFEDVSEEDEPSAADTKEFFNEPNIGSE	F2A F3A	-10+2	U:254-355
Orp2a:At-x2-1D	32	2x 297-328	DGKQEFEDVSEEDEPDFHDTKEFFNEPNIGSE	S1D	-10+2	U:254-355
Fapp2:Md-1E	32	300-331	SPSASQKEEEEEVQEFFSAMNEDFSDIELLED	T1E	-9+1	U:237-336
Fapp2:Md-4D	32	300-331	SPSASQKEEEEEVQTFFDAMNEDFSDIELLED	S4D	-9+1	U:237-336
Fapp2:Md-1E+4D	32	300-331	SPSASQKEEEEEVQEFFDAMNEDFSDIELLED	T1E S4D	-9+1	U:237-336
Fapp2:Hsx2-wt	37	2x 291-327	SDSSCSPECLWEEGKEVIPTFFSTMNTSFSDIELLED	none	-9+1	U:238-331
Fapp2:Hsx2-1E/4D	37	2x 291-327	SDSSCSPECLWEEGKEVIPEFFDTMNTSFSDIELLED	T1E S4D	-9+1	U:238-331
Fapp2:Hsx2-EDA	37	2x 291-327	SDSSCSPECLWEEGKEVIPEFFDAMNTSFSDIELLED	“ “+5TA	-9+1	U:238-331
Fapp2:Hsx4wt	37	4x 291-327	SDSSCSPECLWEEGKEVIPTFFSTMNTSFSDIELLED	none	-9+1	U:238-331
Fapp2:Hsx4-4D	37	4x 291-327	SDSSCSPECLWEEGKEVIPTFFDTMNTSFSDIELLED	S4D	-9+1	U:238-331
MAST205-wt	36	799-834	EFIPQLESEDDTSYFDTRSERYHHMDSEDEEEVSED	none	-12+2	U:796-924
c-Src-5A‡	32	505-536	EPEERPTFEYLQAFLEDYFTATEPQYQPGENL	S5A	-6+1	**H:512-521**
Ypt11p-wt	41	346-386	DLVENGCFENNDPCVSITTSDDVQGHEQEFHDTVEEPFNFT	none	-9+0	H:365-376
Protrudin°		(272-309)	SQDLTPGSVEEAEEAEPDEEFKDAIEETHLVVLEDDEG		-12+0	H:290-296
USP20°		(262-296)	DSDSSDTDEKREGDRSPSEDEFLSCDSSSDRGEGD		-14+4	U:251-442
RMD3°		(149-199)	SDSTGSSSVYFTASSGATFTDAESEGGYTTANAESDNERDSDKESEDGEDE		-15+2	U:128-200

This table provides more detailed information on sequences referred to in Table 1.

† All sequences (°except protrudin, USP20 and RMD3) were cloned after GFP-myc. The accession numbers and species of origin (where not human) of the open reading frames are: Rab3GAP1 - 289547212; AKAP220 - 7671392; AKAP110 - 217416351; Orp1c: *Glycine max* - 164457637; Orp2a: *A. thaliana* - 7269100; Fapp2: *Monodelphis domestica* - 312283618; Fapp2: *Hs* - 158706386; MAST205 - 112363080; Src - 4885609; Ypt11p - 82795261 (*S. cerevisiae*) and protrudin - 50557646. Sequences are coloured with acids red, S/T orange, basic residues blue. The core FFAT-like motifs are highlighted in light blue. Variations introduced for residues in this study are in bold and underlined.

# is the number of amino acids from each target proteins expressed in this study.

* “changes”: summarizes changes introduced to the natural motif. In dimers/tetramers changes were applied to all copies.

¶ “gen.±”: indicates the overall charge (D/E = -1; K/R = +1) in the flanks close to, but excluding, the FFAT-like motif.

§ “structure”: is the predicted (if known then **in bold**) structure in this region of the protein. “U” means unstructured or extended. Numbers refer to the residues involved in any main structural feature.

‡ S5 in this motif was substituted with A to prevent any inhibitory phosphorylation of that site.

° FFAT-like motifs of protrudin, USP20 and RMD3 were not tested in this study, but are shown for comparison. An alternative FFAT-like motif in RMD3 is underlined.
